# Monocyte Transcriptional Responses to Mycobacterium tuberculosis Associate with Resistance to Tuberculin Skin Test and Interferon Gamma Release Assay Conversion

**DOI:** 10.1128/msphere.00159-22

**Published:** 2022-06-13

**Authors:** Jason D. Simmons, Kimberly A. Dill-McFarland, Catherine M. Stein, Phu T. Van, Violet Chihota, Thobani Ntshiqa, Pholo Maenetje, Glenna J. Peterson, Penelope Benchek, Mary Nsereko, Kavindhran Velen, Katherine L. Fielding, Alison D. Grant, Raphael Gottardo, Harriet Mayanja-Kizza, Robert S. Wallis, Gavin Churchyard, W. Henry Boom, Thomas R. Hawn

**Affiliations:** a TB Research & Training Center, Department of Medicine, University of Washington, Seattle, Washington, USA; b Department of Population & Quantitative Health Sciences, Case Western Reserve University, Cleveland, Ohio, USA; c Department of Medicine, Case Western Reserve University, Cleveland, Ohio, USA; d Fred Hutchinson Cancer Research Center, Seattle, Washington, USA; e School of Public Health, University of Witwatersrand, Johannesburg, South Africa; f The Aurum Institute, Parktown, South Africa; g Uganda-CWRU Research Collaboration, Kampala, Uganda; h TB Centre, London School of Hygiene & Tropical Medicine, London, United Kingdom; i Africa Health Research Institute, School of Nursing and Public Health, University of KwaZulu-Natal, Durban, South Africa; j University of Lausanne and Lausanne University Hospital, Lausanne, Switzerland; k Swiss Institute of Bioinformatics, Lausanne, Switzerland; l Department of Medicine, School of Medicine, Makerere University, Kampala, Uganda; m Department of Medicine, Vanderbilt University, Nashville, Tennessee, USA; University of Kentucky

**Keywords:** tumor necrosis factor alpha, innate immunity, sequence analysis, RNA, transcriptome, host-pathogen interactions, *Mycobacterium tuberculosis*

## Abstract

Heavy exposure to Mycobacterium tuberculosis, the etiologic agent of tuberculosis (TB) and among the top infectious killers worldwide, results in infection that is cleared, contained, or progresses to disease. Some heavily exposed tuberculosis contacts show no evidence of infection using the tuberculin skin test (TST) and interferon gamma release assay (IGRA); yet the mechanisms underlying this “resister” (RSTR) phenotype are unclear. To identify transcriptional responses that distinguish RSTR monocytes, we performed transcriptome sequencing (RNA-seq) on monocytes isolated from heavily exposed household contacts in Uganda and gold miners in South Africa after *ex vivo* M. tuberculosis infection. Gene set enrichment analysis (GSEA) revealed several gene pathways that were consistently enriched in response to M. tuberculosis among RSTR subjects compared to controls with positive TST/IGRA testing (latent TB infection [LTBI]) across Uganda and South Africa. The most significantly enriched gene set in which expression was increased in RSTR relative to LTBI M. tuberculosis-infected monocytes was the tumor necrosis factor alpha (TNF-α) signaling pathway whose core enrichment (leading edge) substantially overlapped across RSTR populations. These leading-edge genes included candidate resistance genes (*ABCA1* and *DUSP2*) with significantly increased expression among Uganda RSTRs (false-discovery rate [FDR], <0.1). The distinct monocyte transcriptional response to M. tuberculosis among RSTR subjects, including increased expression of the TNF signaling pathway, highlights genes and inflammatory pathways that may mediate resistance to TST/IGRA conversion and provides therapeutic targets to enhance host restriction of M. tuberculosis intracellular infection.

**IMPORTANCE** After heavy M. tuberculosis exposure, the events that determine why some individuals resist TST/IGRA conversion are poorly defined. Enrichment of the TNF signaling gene set among RSTR monocytes from multiple distinct cohorts suggests an important role for the monocyte TNF response in determining this alternative immune outcome. These TNF responses to M. tuberculosis among RSTRs may contribute to antimicrobial programs that result in early clearance or the priming of alternative (gamma interferon-independent) cellular responses.

## INTRODUCTION

Aerosol exposure to Mycobacterium tuberculosis results in three basic outcomes in humans: (i) failure of the bacterium to establish infection or its early clearance, (ii) infection within alveolar macrophages and other myeloid cells that is contained but not sterilized by an adequate host cellular response, or (iii) progressive pulmonary or disseminated infection that results in human disease and potentially ongoing transmission. The critical host-pathogen events that result in sterilizing immunity are largely unknown.

To better understand the immunologic mechanism of natural resistance to M. tuberculosis infection, we (1) and others (2–4) have profiled immune cell signatures from individuals who fail to convert the tuberculin skin test (TST) and interferon gamma (IFN-γ) release assay (IGRA) despite heavy exposure to M. tuberculosis, which we label the “resister” (RSTR) phenotype (5–7). We examined household contacts of pulmonary tuberculosis (TB) cases in Uganda who completed serial TST and IGRA testing over 8 to 10 years of follow-up and male gold miners in South Africa who have extreme occupational M. tuberculosis exposure (8–10). Resistance to TST/IGRA conversion in RSTRs may be explained by multiple mechanisms, including early clearance through innate immune responses (2, 11), IFN-γ-independent T cell responses (1, 12), or humoral responses (1, 3, 5, 6). However, we do not currently know whether RSTR subjects have distinct monocyte/macrophage responses upon M. tuberculosis infection and whether the cellular inflammatory response is heightened or diminished compared to latent TB infection (LTBI) subjects.

We recently compared RSTR and LTBI transcriptomes in whole blood and in monocytes to identify any cellular pathways that may distinguish these clinical phenotypes or provide clues into their unique immune responses (13). Across heterogeneous cohorts, RSTR individuals had strong enrichment of gene sets in monocyte metabolic pathways. However, these studies were performed in the absence of any stimulation of M. tuberculosis antigens or live infection. In the current study, we compare the monocyte transcriptional responses between RSTR and LTBI subjects following *ex vivo* M. tuberculosis infection. We discovered multiple pathways that distinguish these clinical phenotypes across study sites. Surprisingly, we detected increased expression of genes involved with tumor necrosis factor (TNF) signaling among RSTR monocytes, which may contribute to the unique immunologic outcomes to M. tuberculosis infection in these highly exposed cohorts.

## RESULTS

### Inflammatory and metabolic gene set enrichment distinguishes RSTR transcriptomes across populations.

**FIG 1:**
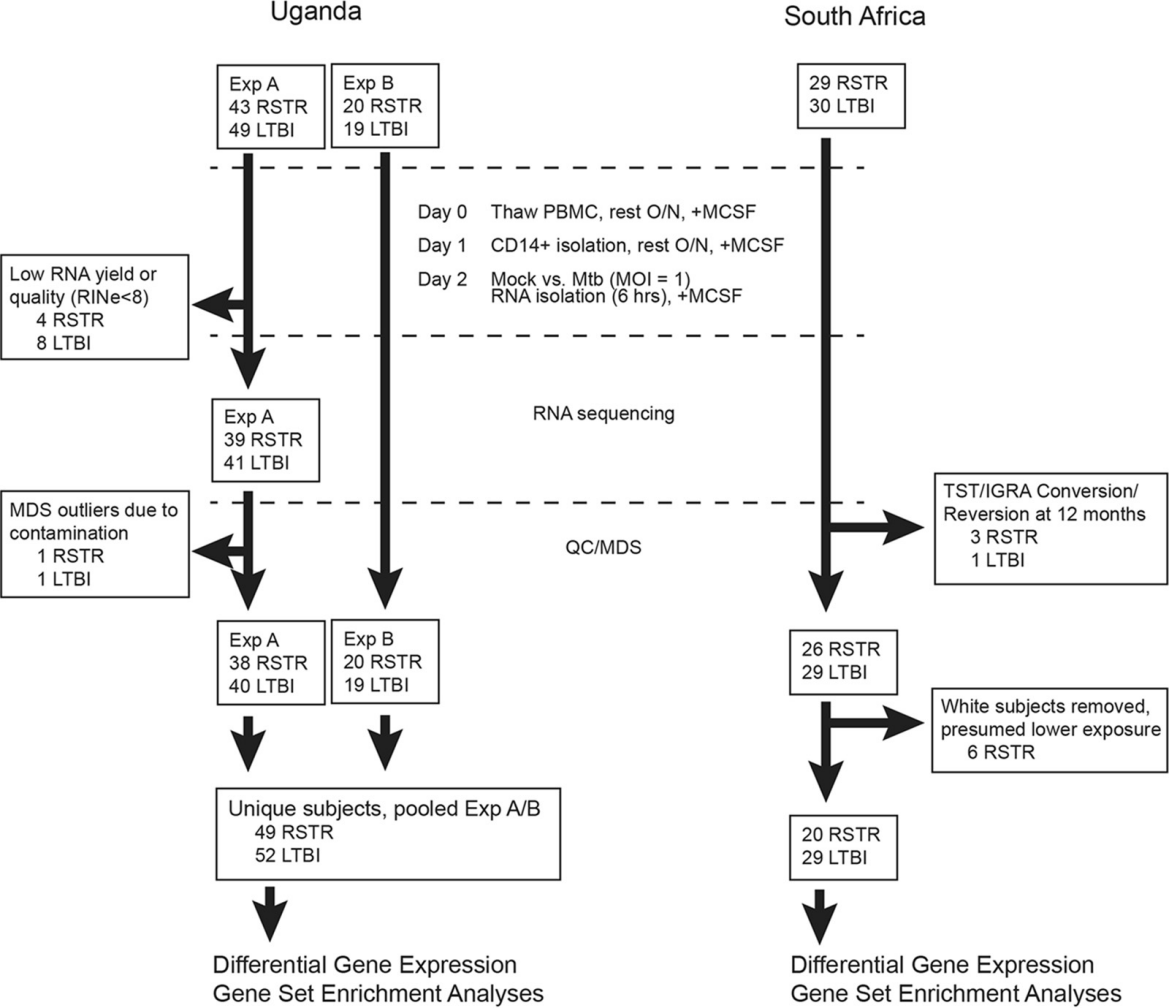
Uganda household contact and South Africa gold miner recruitment and monocyte RNA-seq analyses. Peripheral blood mononuclear cells from each cohort were thawed and rested in M-CSF overnight before CD14^+^ magnetic bead column isolation, plated to allow for adherence overnight, and then infected with M. tuberculosis (H37Rv) for 6 h (MOI, 1). RNA was isolated for library preparation and RNA sequencing. The indicated subjects were excluded due to failed RNA yield or quality and contamination in Uganda. In South Africa, additional samples were excluded due to TST/IGRA conversion or reversion (3 RSTR and 1 LTBI subjects) and based on ancestry related to presumed lower M. tuberculosis exposure (6 RSTR subjects). The experiment was performed twice (Exp A and Exp B) among partially overlapping subjects from Uganda, resulting in a final 101 unique subjects (49 RSTR and 52 LTBI subjects) and once from 49 South Africa gold miner subjects (20 RSTR and 29 LTBI subjects). QC/MDS, quality control/multidimensional scaling.

To evaluate whether resistance to TST/IGRA conversion correlates with distinct monocyte transcriptional responses to M. tuberculosis infection, we examined two cohorts of HIV-negative subjects with heavy M. tuberculosis exposure in Uganda and South Africa and compared transcriptomes from those with persistently negative TST/IGRA (RSTR) versus LTBI controls with concordant positive tests. We enriched CD14^+^ monocytes from cryopreserved peripheral blood mononuclear cells (PBMCs) from each cohort (*n* = 49 RSTR and 52 LTBI subjects in Uganda; *n* = 20 RSTR and 29 LTBI subjects in South Africa) and infected them with M. tuberculosis (H37Rv) or medium for 6 h before transcriptome (RNA-seq) analysis (Fig. 1). No demographic or epidemiologic features were associated with RSTR or LTBI phenotypes in Uganda (see Table S1 in the supplemental material), whereas ancestry was associated with the RSTR phenotype in South Africa (Table S2). We stratified subsequent South Africa analyses according to the dominant ethnic group.

10.1128/msphere.00159-22.7 TABLE S1 Comparison of demographic and epidemiologic characteristics of Ugandan subjects. Download Table S1, DOCX file, 0.02 MB. Copyright © 2022 Simmons et al. 2022 Simmons et al. https://creativecommons.org/licenses/by/4.0/ This content is distributed under the terms of the Creative Commons Attribution 4.0 International license.

10.1128/msphere.00159-22.8 TABLE S2 Comparison of demographic and epidemiologic characteristics of South African subjects. Download Table S2, DOCX file, 0.02 MB. Copyright © 2022 Simmons et al. 2022 Simmons et al. https://creativecommons.org/licenses/by/4.0/ This content is distributed under the terms of the Creative Commons Attribution 4.0 International license.

**FIG 2:**
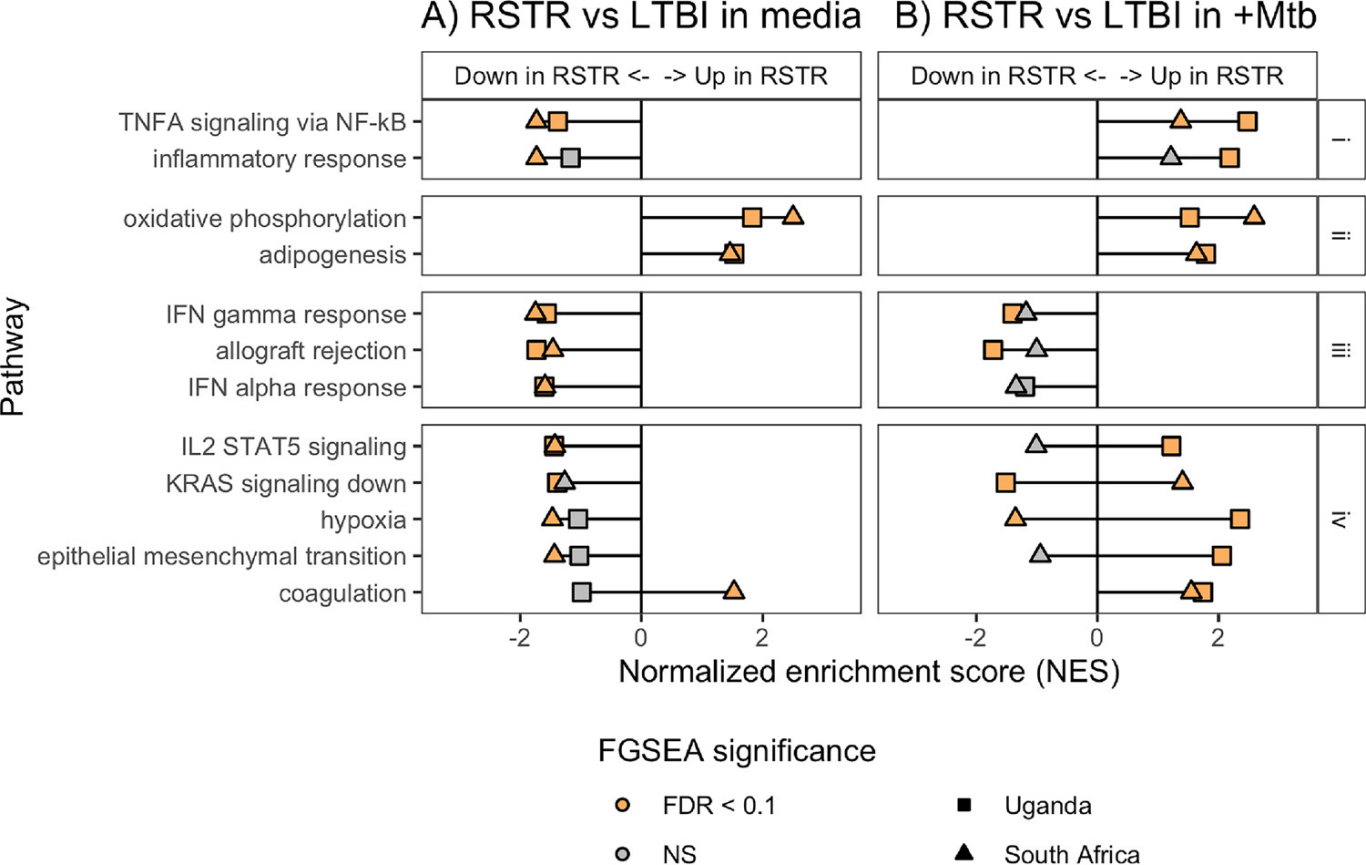
Gene set enrichment analysis identifies pathways that distinguish RSTR and LTBI phenotypes under unstimulated and M. tuberculosis-infected conditions. RSTR and LTBI global transcriptional differences under unstimulated (A) or M. tuberculosis-infected (B) conditions were probed using FGSEA and “hallmark” curated gene sets (MSigDB). The effects of M. tuberculosis on global expression differences were similarly compared (Fig. S1). Gene sets that were significantly enriched (FDR, <0.1) both by phenotype (A and B) and by stimulation condition (Fig. S1A and B) across Uganda and South Africa are shown. Global expression patterns for each gene set can be grouped by direction of enrichment across analyses (i to iv), most of which are highly concordant across clinical sites (i to iii). For each analysis, a normalized enrichment score (NES) is plotted according to the direction of enrichment for the Uganda (square) and South Africa (triangle) data sets, with color indicating significant enrichment (FDR, <0.1). NS, nonsignificant (FDR, ≥0.1).

Using gene set enrichment analysis (GSEA) (14, 15), we identified multiple “hallmark” gene sets (16) that differentiated RSTR and LTBI monocytes across Uganda and South Africa sites. These enriched RSTR transcriptional signatures included metabolic and inflammatory gene sets among unstimulated monocytes (Fig. 2A), a finding that we reported previously (13). Upon M. tuberculosis stimulation, several gene expression patterns emerged (groups i to iv in Fig. 2B). First (group i), genes within the “*TNFα* signaling via NF-κB” (*TNFα* hallmark) and the “inflammatory response” hallmark gene sets had lower expression at baseline and higher expression following M. tuberculosis among RSTR than LTBI monocytes. Second (group ii), collective expression of the “oxidative phosphorylation” and “adipogenesis” gene sets remained higher among RSTR subjects regardless of stimulation. M. tuberculosis infection resulted in decreased expression of these metabolic gene sets in both RSTR and LTBI (Fig. S1), consistent with a shift away from oxidative phosphorylation and toward aerobic glycolysis (Warburg effect) induced by mycobacterial stimuli (17, 18), but the relative RSTR versus LTBI expression patterns suggest that RSTR monocytes are less susceptible to this shift. Third (group iii), collective expression of genes in the “*IFNγ* response” gene set was lower in RSTR (versus LTBI) regardless of M. tuberculosis stimulation. At least for the M. tuberculosis-infected condition, this finding is expected, considering our phenotypes are defined by an M. tuberculosis-specific IFN-γ response (i.e., IGRA). The “allograft rejection” and “*IFNα* response” gene sets also followed this pattern. Overlap of the IFN-α and IFN-γ leading-edge (LE) genes (Fig. S2), which is a subset of genes that contributes to the core enrichment of a gene set (15), suggests that the IFN-α enrichment is largely driven by IFN-γ signaling.

10.1128/msphere.00159-22.2 FIG S1 Gene set enrichment analysis identifies pathways that distinguish medium- and M. tuberculosis-stimulated conditions in LTBI and RSTR monocytes. Global transcriptional differences (medium versus M. tuberculosis stimulation) in LTBI (A) and RSTR (B) monocytes were probed using fast gene set enrichment analysis (FGSEA) and “hallmark” curated gene sets (molecular signatures database [MSigDB]). We restricted this analysis to gene sets that were significantly enriched (FDR, <0.1) by both the stimulation condition shown here and by phenotype (Fig. 2) across Uganda and South Africa. These enriched gene sets are grouped as described in the legend to Fig. 2. For each analysis, the normalized enrichment score (NES) is plotted according to the direction of enrichment for Uganda (square) and South Africa (triangle) data sets, with color indicating significant enrichment (FDR, <0.1). FDR, false-discovery rate; NS, nonsignificant (FDR, ≥0.1). Download FIG S1, TIF file, 2.7 MB. Copyright © 2022 Simmons et al. 2022 Simmons et al. https://creativecommons.org/licenses/by/4.0/ This content is distributed under the terms of the Creative Commons Attribution 4.0 International license.

10.1128/msphere.00159-22.3 FIG S2 Core enrichments of IFN-γ and IFN-α gene sets among LTBI transcriptomes overlap and predominately represent the IFN-γ response. The number of genes in the core enrichment (leading edge) are shown for the Uganda FGSEA analyses using either the “*IFNγ* signaling” (A, B) or “*IFNα* signaling” (C, D) hallmark gene set under either the medium-stimulated (A, C) or M. tuberculosis-stimulated (B, D) condition. For each analysis, the percentage of leading-edge genes unique to the *IFNγ* signaling gene set compared to the *IFNa* signaling gene set is shown. The greater number of unique genes in the *IFNγ* signaling gene set suggests that the major contribution toward the *IFNα* signaling enrichment is driven by IFN-γ signaling. Download FIG S2, TIF file, 0.6 MB. Copyright © 2022 Simmons et al. 2022 Simmons et al. https://creativecommons.org/licenses/by/4.0/ This content is distributed under the terms of the Creative Commons Attribution 4.0 International license.

**FIG 3:**
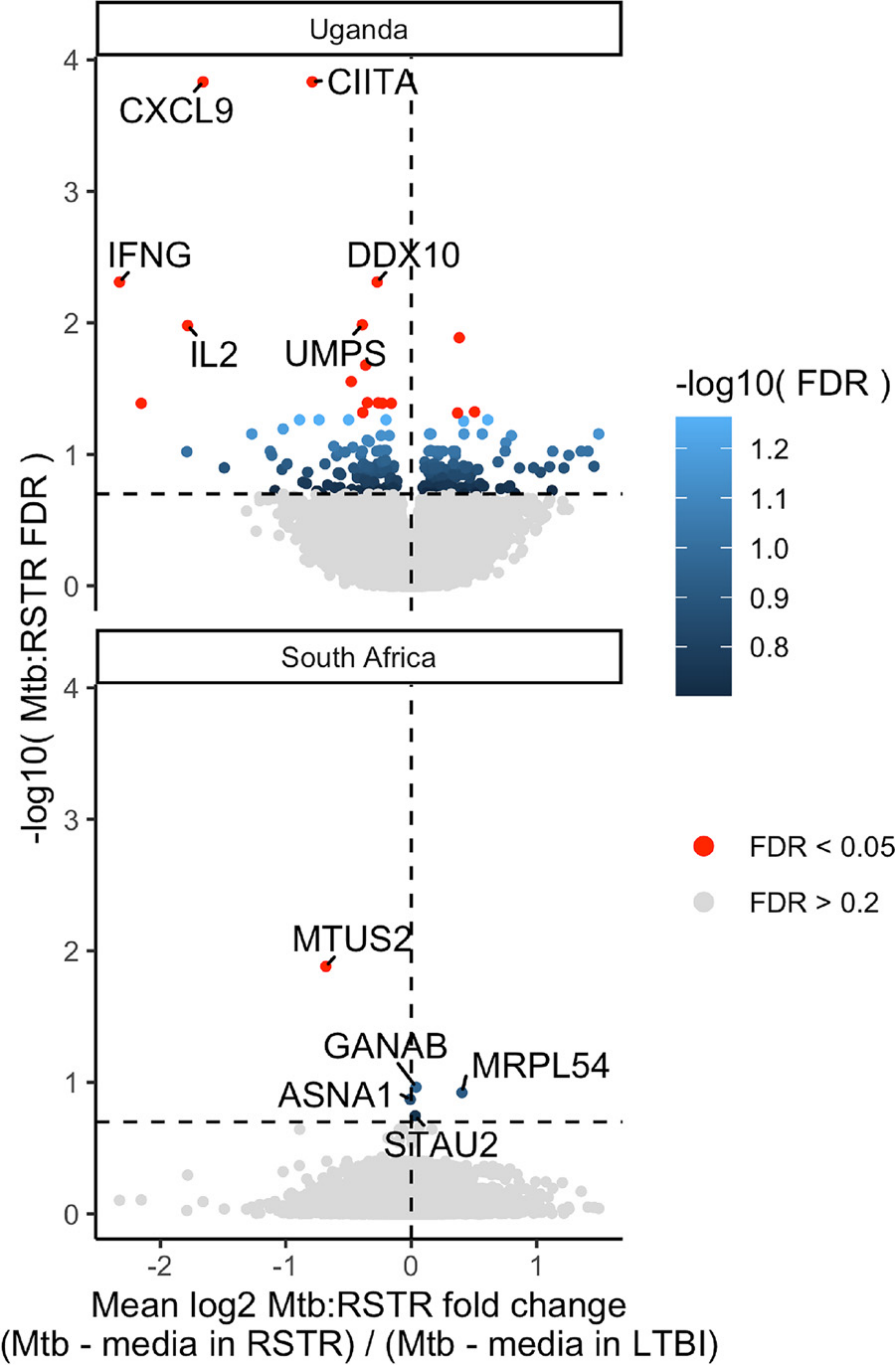
Differentially expressed genes distinguish RSTR and LTBI phenotypes, but are not shared across Uganda and South Africa. Differentially expressed genes were identified using an interaction model that incorporates an interaction term (“Mtb:RSTR”) in addition to the main effects phenotype (RSTR versus LTBI) and stimulation (medium versus M. tuberculosis). Using this model, 260 DEGs were identified in Uganda and 5 were identified in South Africa (FDR, <0.2). Volcano plots for the Uganda (top) and South Africa (bottom) analysis indicate changes in gene expression in response to M. tuberculosis stimulation (M. tuberculosis versus medium, log_2_ fold change) that contrast RSTR and LTBI.

**FIG 4:**
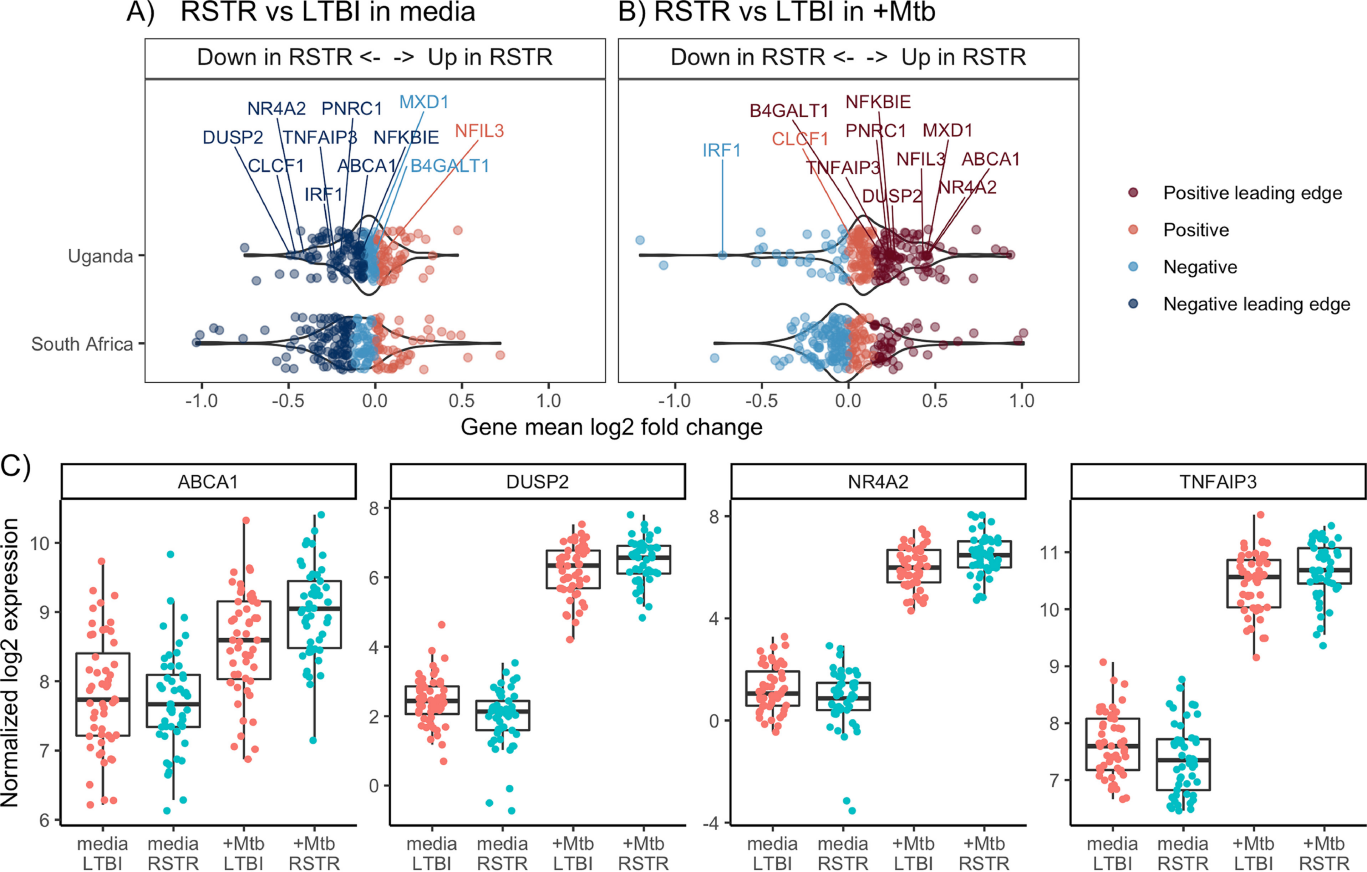
Core Uganda enrichment of TNF-α signaling gene set contains multiple differentially expressed genes. Mean relative expression (log_2_ fold change) between RSTR and LTBI is plotted for each gene in the “*TNFα* response via NF-κB” hallmark gene set using pairwise contrasts for the medium-stimulated (A) and M. tuberculosis-stimulated (B) conditions for Uganda (top) and South Africa (bottom) monocyte analyses. Leading-edge genes are indicated by darker dots. Gene labels reflect DEGs identified in Uganda from the interaction model that incorporates main effects and the stimulation:phenotype term. Gene label shading indicates a DEG that is also a leading-edge member (dark label) versus DEGs that did not contribute this core enrichment (light label). (C) Box plots for select Uganda DEGs *ABCA1*, *DUSP2*, *NR4A2*, and *TNFAIP3*, each of which contributed to the GSEA core enrichment are shown: the median (line), interquartile range (box), and 1.5× interquartile range (whiskers) of normalized expression (log_2_) for each subject (dot) were plotted.

Since RSTR monocytes have greater upregulation of the *TNFα* hallmark and inflammatory response pathways in response to M. tuberculosis infection, we hypothesized that phenotype-specific modulation of these pathways is involved in resistance to TST/IGRA conversion. We first looked for candidate resistance genes that were differentially expressed among RSTRs at each study site. Using an interaction model, which includes an interaction term between the M. tuberculosis infection (“M. tuberculosis group” versus “medium”) and clinical phenotype (RSTR versus LTBI) main effects, we identified 260 differentially expressed genes (DEGs) in Uganda and five DEGs in South Africa (false-discovery rate [FDR], <0.2) (Fig. 3; Tables S3 and S4). Principal-component analysis showed clustering by infection status and not phenotype (Fig. S3), and *post hoc* pairwise contrasts of these DEGs indicated expression differences between RSTR and LTBI phenotypes were largely restricted to the M. tuberculosis-stimulated condition (Fig. S4). However, none of these candidate resistance genes overlapped across sites. To identify candidate RSTR genes shared across populations, we next explored gene-level features of the *TNFα* hallmark pathway limiting the analysis to genes that either were DEGs or were included in the leading-edge subset that defines the core enrichment from each GSEA (Fig. 4A and B). The leading-edge subsets showed substantial overlap across Uganda and South Africa with consistent enrichment under the medium-stimulated condition (RSTR expression decreased), M. tuberculosis-stimulated condition (RSTR expression increased), or both (Table 1). Interestingly, *TNF* was neither differentially expressed nor a member of the overlapping leading-edge subset across populations, suggesting the enrichment of *TNFα* hallmark genes among RSTRs is not strictly a function of *TNF* expression. Six Uganda DEGs were leading-edge members under both the medium- and M. tuberculosis-stimulated conditions, including *ABCA1*, *DUSP2*, *NR4A2*, and *TNFAIP3* (Fig. 4C). Overall, our results identify RSTR genes related to TNF-α signaling, yet distinct from *TNF*, that we hypothesize play important roles in mediating resistance to TST/IGRA conversion.

**TABLE 1:** Overlap of *TNFα* gene hallmark leading-edge subsets

Gene in subset^*a*^			
Medium (RSTR down)		M. tuberculosis (RSTR up)	
Uganda (33/98 LE genes)	South Africa (35/92 LE genes)	Uganda (37/96 LE genes)	South Africa (12/47 LE genes)
*CCL20*	*CCL20*	*CCL20*	*CCL20*
*IL7R*	*IL7R*	*IL7R*	*IL7R*
*INHBA*	*INHBA*	*INHBA*	*INHBA*
*PTX3*	*PTX3*	*PTX3*	*PTX3*
*TNFAIP6*	*TNFAIP6*	*TNFAIP6*	*TNFAIP6*
*TNIP1*	*TNIP1*	*TNIP1*	*TNIP1*
***ABCA1***	*ABCA1*	***ABCA1***	
*AREG*	*AREG*	*AREG*	
*CCL4*	*CCL4*	*CCL4*	
*CXCL1*	*CXCL1*	*CXCL1*	
*CXCL2*	*CXCL2*	*CXCL2*	
*CXCL3*	*CXCL3*	*CXCL3*	
***DUSP2***	*DUSP2*	***DUSP2***	
*DUSP4*	*DUSP4*	*DUSP4*	
*EHD1*	*EHD1*	*EHD1*	
*GPR183*	*GPR183*	*GPR183*	
*HES1*	*HES1*	*HES1*	
*IFIT2*	*IFIT2*	*IFIT2*	
	*IL1B*	*IL1B*	*IL1B*
*IL23A*	*IL23A*	*IL23A*	
	*MARCKS*	*MARCKS*	*MARCKS*
*NFKB2*	*NFKB2*	*NFKB2*	
*NFKBIA*	*NFKBIA*	*NFKBIA*	
*NR4A1*	*NR4A1*	*NR4A1*	
***NR4A2***	*NR4A2*	***NR4A2***	
*OLR1*		*OLR1*	*OLR1*
*PDE4B*	*PDE4B*	*PDE4B*	
*PFKFB3*	*PFKFB3*	*PFKFB3*	
***PNRC1***	*PNRC1*	***PNRC1***	
	*PTGS2*	*PTGS2*	*PTGS2*
	*SLC2A6*	*SLC2A6*	*SLC2A6*
*SOCS3*	*SOCS3*	*SOCS3*	
*SOD2*	*SOD2*	*SOD2*	
*TNC*		*TNC*	*TNC*
***TNFAIP3***	*TNFAIP3*	***TNFAIP3***	
*ZC3H12A*	*ZC3H12A*	*ZC3H12A*	
*ZFP36*	*ZFP36*	*ZFP36*	

a Genes are listed if they were members of the leading-edge (LE) subset in at least 3 of 4 gene set enrichment analyses (columns) using the hallmark “*TNF*α signaling via NF-κB” gene set (MsigDB). Expression of these LE genes was lower (medium condition) or higher (M. tuberculosis condition) in RSTR than in LTBI monocytes. Of the 200 genes in this gene set, the LE subset consisted of 98 genes (Uganda medium), 92 genes (South Africa medium), 96 genes (Uganda M. tuberculosis), and 47 genes (South Africa M. tuberculosis) in the respective analyses. Gene order is alphabetical, and genes are grouped by overlap across all 4 analyses (above line) or 3 of 4 analyses (below line). Boldface genes are included among the 260 DEGs in Uganda (interaction model).

10.1128/msphere.00159-22.4 FIG S3 M. tuberculosis infection, and not clinical phenotype, results in greater monocyte transcriptional differences. The first two principal components of global transcription of each sample from Uganda (A, B) and South Africa (C, D) are plotted and reveal two clusters that clearly separate the monocyte stimulation condition (A, C) but not clinical phenotype (B, D). Download FIG S3, TIF file, 1.2 MB. Copyright © 2022 Simmons et al. 2022 Simmons et al. https://creativecommons.org/licenses/by/4.0/ This content is distributed under the terms of the Creative Commons Attribution 4.0 International license.

10.1128/msphere.00159-22.5 FIG S4 M. tuberculosis infection results in greater differential gene expression between RSTR and LTBI phenotypes. Differentially expressed genes (DEGs) that were identified using an interaction model (FDR, <0.2) (Fig. 3) were further analyzed using *post hoc* pairwise contrasts in Uganda (top) and South Africa (bottom) according to each phenotype or stimulation condition (medium versus M. tuberculosis or RSTR versus LTBI). A single gene was differentially expressed in RSTR and LTBI subjects in South Africa when M. tuberculosis stimulation was not considered and accordingly was also included in the *post hoc* pairwise contrast analysis (260 DEGs from Uganda and 6 DEGs from South Africa, indicated in red). All genes remained significant for at least one contrast (FDR, <0.2) although differential gene expression largely reflected the M. tuberculosis-stimulated condition. Mtb*RSTR interaction model, expression model including main effects stimulation (M. tuberculosis versus medium) + phenotype (RSTR versus LTBI) + interaction term (Mtb:RSTR) + covariates; Mtb:RSTR contrast model, expression model Mtb:RSTR pairwise comparisons + covariates. Download FIG S4, TIF file, 1.1 MB. Copyright © 2022 Simmons et al. 2022 Simmons et al. https://creativecommons.org/licenses/by/4.0/ This content is distributed under the terms of the Creative Commons Attribution 4.0 International license.

10.1128/msphere.00159-22.9 TABLE S3 Mtb*RSTR differentially expressed genes (DEGs). Shown are Excel worksheets. Download Table S3, XLSX file, 0.02 MB. Copyright © 2022 Simmons et al. 2022 Simmons et al. https://creativecommons.org/licenses/by/4.0/ This content is distributed under the terms of the Creative Commons Attribution 4.0 International license.

10.1128/msphere.00159-22.10 TABLE S4 Uganda Mtb*RSTR DEG polymorphisms associate with clinical phenotype. Download Table S4, DOCX file, 0.02 MB. Copyright © 2022 Simmons et al. 2022 Simmons et al. https://creativecommons.org/licenses/by/4.0/ This content is distributed under the terms of the Creative Commons Attribution 4.0 International license.

### Polymorphisms in Uganda DEGs associate with the RSTR phenotype.

Genetic variations at multiple chromosomal loci have previously been associated with resistance to TST conversion (19–23). We next used a candidate gene association study to examine whether single nucleotide polymorphisms (SNPs) of ≤5 kb *cis* to the 260 DEGs were associated with the RSTR clinical phenotype among Ugandan participants (*n *= 74 RSTR and 189 LTBI subjects). Among these 5,248 examined SNPs, we identified 11 SNPs in 10 genes that associated with the RSTR or LTBI phenotype (*P* < 0.005) (Table S4). These 10 genes included *CIITA* (rs6498130), which was also a leading DEG (log_2_ fold change, −0.79; FDR, 1.5E−04), for which expression increased in LTBI monocytes but decreased in RSTR monocytes following M. tuberculosis stimulation (Fig. S5). Among the other associated SNPs, those for *EPB41L3* (rs1719945), *ZNF184* (rs1883216), *AKT3* (rs12144559), and *KLHL29* (rs1530045) are known expression quantitative trait loci (*cis*-eQTL) in various tissues represented in a public database (GTExPortal; https://www.gtexportal.org). This supports a functional role for the proteins encoded by these genes. None of the 5,248 SNPs queried was significant after correction for false discovery (FDR, <0.2), and while this method may be overly stringent considering linkage disequilibrium reduces the actual number of total comparisons, these SNP associations require confirmation.

10.1128/msphere.00159-22.6 FIG S5 Normalized gene expression among Uganda Mtb*RSTR DEGs. Box plots represent normalized log_2_ expression of the indicated genes in each subject (dot) as the median (bold line), interquartile range (box), and 1.5× interquartile ranges (whiskers). Among the top DEGs are T cell cytokine genes (*IFNG* and *IL2*) and IFN-γ-stimulated genes, for which expression increases in the M. tuberculosis-stimulated state specifically among LTBI monocytes (A). *CIITA* is a central transcriptional regulator of other genes, including *BTN2A2* (B), and while these genes are known to also be upregulated in the IFN-γ response, the expression patterns are distinct from those in panel A and suggest unique regulation among RSTR subjects. Download FIG S5, TIF file, 1.5 MB. Copyright © 2022 Simmons et al. 2022 Simmons et al. https://creativecommons.org/licenses/by/4.0/ This content is distributed under the terms of the Creative Commons Attribution 4.0 International license.

### Networks of Uganda DEGs highlight TNF and inflammatory signaling pathways.

**FIG 5:**
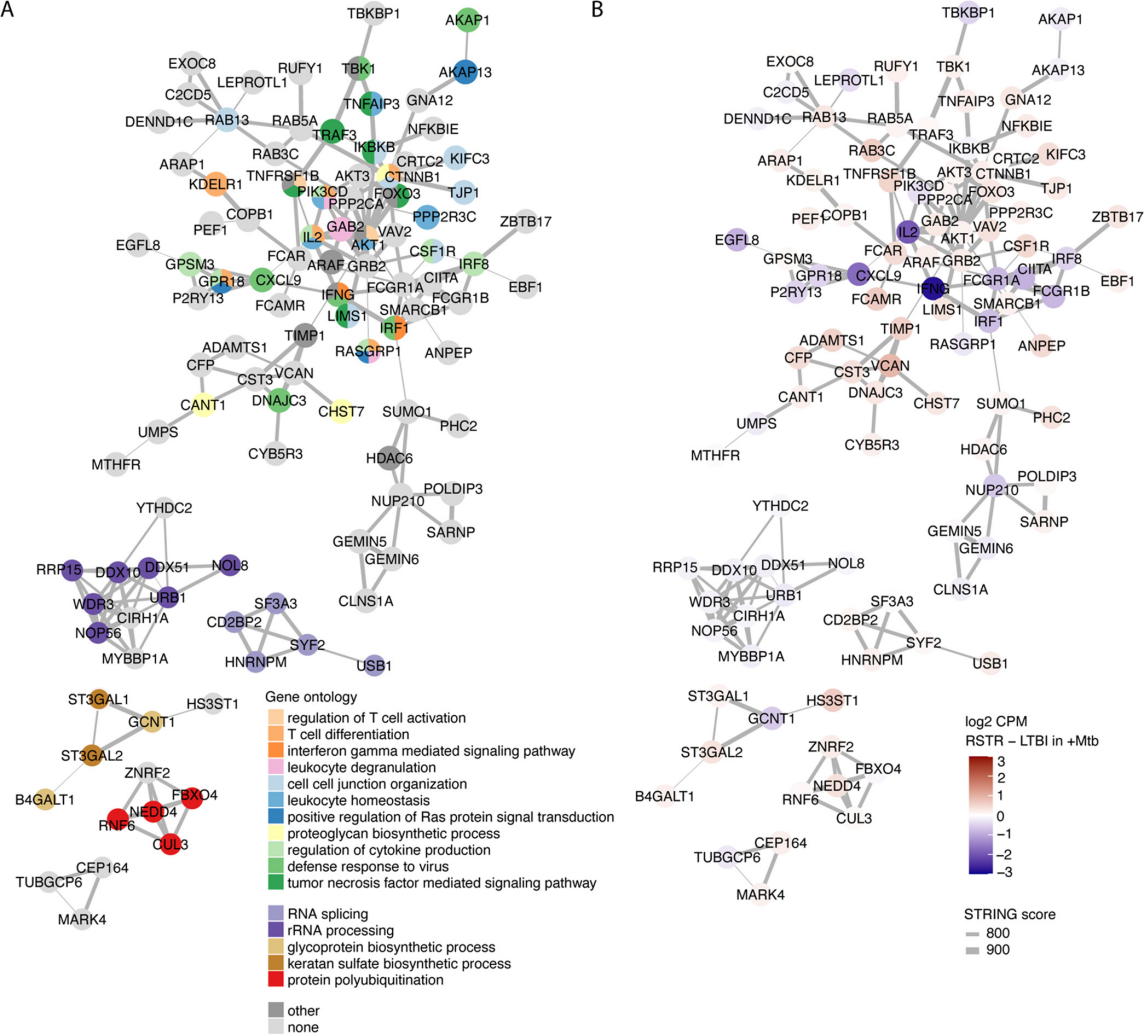
Network analysis of Uganda DEGs identify multiple biologic pathways, including TNF and IFN-γ signaling, that correlate with RSTR and LTBI relative expression. Six gene networks among the 260 Uganda DEGs were identified by STRING. To identify biologic functions of these gene networks, we used topGO enrichment analysis to separately analyze the large network (*n* = 77 DEGs) and each smaller network of 3 or more genes individually. Significantly enriched gene sets (FDR, <0.05) for each cluster were identified using Fisher’s exact test and plotted in topGO, and Gene Ontology (GO) terms were selected from terminal or near-terminal branches of the GO hierarchical networks to capture the most specific GO terms and reduce redundancy. Each DEG was then colored according to its most specific (lowest on tree) enriched GO terms (A) or according to log_2_ fold change (RSTR versus LTBI) expression values in M. tuberculosis-stimulated monocytes (B).

Several DEGs in Uganda with the largest fold change expression values are related to proinflammatory signaling, including *CIITA*, *CXCL9*, *IFNG*, and *IL-2* (Fig. 3; Fig. S5). To explore biologic pathways that relate to these and the other 260 Uganda DEGs, we used STRING network analysis to assign connections among the DEGs that are included in the STRING database (https://string-db.org). We found a large cluster (*n* = 77 genes) of highly interconnected DEGs, five smaller networks, and 155 DEGs that had two or fewer connections (Fig. 5A). To assign biologic function to each network, we used topGO enrichment analysis, where DEGs were labeled according to terminal branches of the hierarchical Gene Ontology (GO) network. The core of the large DEG network related to regulation of cytokine production, TNF signaling, and IFN-γ signaling. Furthermore, this core included *CIITA* and *AKT3*, each of which has SNPs that independently associated with clinical phenotype. Additional subclusters within this network showed enrichment for leukocyte degranulation/homeostasis, cell-cell junction organization and other GO terms. When the STRING networks are labeled by relative expression (RSTR versus LTBI), the IFN-γ subcluster clearly shows higher expression among LTBI monocytes compared to RSTR monocytes following M. tuberculosis stimulation (e.g., *IL2*, *IFNG*, and *CXCL9*), whereas DEGs in the remaining subclusters, including TNF signaling and leukocyte degranulation, have higher expression among RSTR monocytes (e.g., *TNFAIP3*, *TNFRSF1B*, *FOXO3*, and *LIMS1*) (Fig. 5B). The smaller networks were enriched for GO terms related to RNA and rRNA processing, glycoprotein synthesis, and protein polyubiquitination. One network included *ST3GAL1*, which also had an SNP associated with clinical phenotype, but generally the genes in these small networks had modest expression differences between RSTR and LTBI phenotypes. Overall, these DEG networks reinforce conclusions from our global gene set enrichment analysis demonstrating transcriptional differences between RSTR and LTBI monocytes that include elevated expression of IFN-γ signaling among LTBI donors whereas other inflammatory pathways, including TNF, have higher expression among RSTR monocytes following M. tuberculosis infection.

## DISCUSSION

The immunologic mechanisms underlying resistance to TST/IGRA conversion after heavy exposure to M. tuberculosis remain poorly defined. By leveraging rare patient cohorts with rigorous epidemiologic characterization, our study explores pathways enriched across RSTR subjects from two distinct populations. We identify differentially expressed genes and pathways that distinguish transcriptional responses to M. tuberculosis in RSTR and LTBI monocytes. Our results highlight differences related to inflammatory signaling pathways, including the TNF response, which surprisingly was enriched with higher expression in M. tuberculosis-infected RSTR monocytes than in LTBI controls.

TNF activates antimicrobial programs in M. tuberculosis-infected macrophages. TNF is essential for protection from M. tuberculosis disease in mice (24), and the use of multiple anti-TNF biologic agents is associated with an increased risk for TB disease in humans (25–27). Whether this TNF-mediated protection from disease extends to a protective effect at the time of exposure, as might be measured in RSTR subjects, is not currently known. In a previous study of Indonesian household contacts followed for 14 weeks, those who remained IGRA negative (termed “early clearers”) had increased TNF and proinflammatory cytokine responses after *ex vivo* whole-blood stimulation compared to IGRA converters, suggesting that heightened proinflammatory responses at early times postexposure may distinguish RSTR subjects from those who become infected (2). Genetic studies have also associated the TNF response with resistance to TST conversion. Genome-wide linkage studies identified a locus on chromosome 11p14 (*TST1*) associated with TST negativity in regions of high (19) and low (20) endemicity for tuberculosis. This locus was later found to be a quantitative trait locus (QTL) for TNF secretion in response to Mycobacterium bovis BCG (28) and supports the hypothesis that RSTR leukocytes have a distinct TNF response to mycobacterial exposure. TNF may also arise from antigen-stimulated T cells and contribute to IFN-γ-independent cellular immunity that may characterize the RSTR phenotype (6); however, these TNF responses were more likely among LTBI compared to RSTR subjects in Uganda (1). An important distinction from these prior studies is that *TNF* was not a DEG in either population. Accordingly, it is unlikely the protective RSTR response is explained solely by elevated *TNF* expression by myeloid cells following M. tuberculosis infection; rather, we hypothesize that other *TNFα* hallmark genes contribute to resistance to TST/IGRA conversion.

Several DEGs were leading-edge members in the Uganda *TNFα* hallmark GSEA, both under unstimulated (RSTR expression down) and under M. tuberculosis-stimulated (RSTR expression up) conditions. Accordingly, the cellular pathways in which *DUSP2*, *ABCA1*, and *NR4A2* participate may distinguish RSTR and LTBI functions by modulating intracellular M. tuberculosis infection. Dual-specificity phosphatase 2 (PAC-1), encoded by *DUSP2*, is induced by Toll-like receptor stimulation and associated with proinflammatory (29) and anti-inflammatory phenotypes (30) that are mediated through its negative regulation of mitogen-activated protein kinase (MAPK) and STAT3 pathways in macrophages and T cells. Transcriptional analysis of M. tuberculosis-infected macrophages (31, 32) previously identified *ABCA1* as one of the most transcriptionally upregulated genes, which may relate to its role as a cholesterol efflux pump located at the plasma and endosomal membranes, where it could regulate M. tuberculosis cholesterol uptake (32) and is linked to the IFN-γ response (31). Finally, *NR4A2* is a nuclear receptor and early response gene that is upregulated by lipopolysaccharide (LPS), cytokines, and oxidized lipids (33), and as a transcription factor, it has pleotropic activities, including the regulation of macrophage polarity (34) and fatty acid metabolism (35). Further studies are required to establish a role for these gene pathways in modulating intracellular M. tuberculosis replication or resistance to TST/IGRA conversion, and if validated, they may provide potential targets for therapeutic intervention or the development of biomarkers for M. tuberculosis exposure.

In contrast to the increase in M. tuberculosis-stimulated expression of *TNFα* hallmark (and inflammatory response) gene sets among RSTR subjects, expression of the *IFNγ* response and *IFNα* response gene sets remained lower in RSTR subjects in unstimulated and M. tuberculosis-infected monocytes. In Gambian subjects who were followed for 3 months with TST/IGRA testing after household M. tuberculosis exposure, an increased type I IFN response among IGRA nonconverters was detected using transcriptomic profiling of unstimulated whole blood (3). The discordance in the type I IFN enrichment in these findings may relate to the difference in specimens (whole blood versus monocytes) and differences in study design, such as the timing of household exposure, which was recent among Gambian contacts and either remote (Uganda) or undefined (South Africa) in our analysis relative to blood collection. Alternatively, considering most genes in the *IFNα* response core enrichment are also members of the *IFNγ* hallmark gene set (see Fig. S2 in the supplemental material), it is likely the enrichment of these pathways among LTBI transcriptomes primarily reflects the IFN-γ response.

Both the global monocyte IFN-γ gene set enrichment and the identification of DEGs related to IFN-γ or IL-2 signaling (e.g., *CIITA*, *IFNG*, *CXCL9*, and *IL2*) were identified among LTBI monocytes. *CXCL9* expression in whole blood was also found to be increased at baseline in Gambian subjects who ultimately converted TST after household exposure (3). As T lymphocyte cytokines, increases in *IFNG* and *IL2* transcripts in M. tuberculosis-stimulated LTBI monocyte cultures may result from lymphocyte impurity in our monocyte cultures, but several alternative considerations should be made. First, the short stimulation (6 h) may restrict the breadth or magnitude of any paracrine effects due to IFN-γ secretion by lymphocytes. Second, since the IFN-γ gene set was significantly enriched in unstimulated LTBI monocytes, differences between LTBI and RSTR transcriptional programs are not solely a function of the IGRA-based categorical phenotype definitions that require the presence of M. tuberculosis antigens. Third, a distinction among these top DEGs should be made with respect to *CIITA*, which encodes a polymorphism (rs6498130) that independently associated with the RSTR phenotype in Uganda and recently was linked to M. tuberculosis susceptibility (36). Unlike other top DEGs involved in the T cell response (*IFNG*, *IL2*, and *CXCL9*) where expression increased following M. tuberculosis stimulation largely in LTBI monocytes (Fig. S5A), *CIITA* expression decreased following M. tuberculosis infection specifically in RSTR monocytes. As a key transcriptional cofactor for major histocompatibility complex class II (MHC-II) expression, the decreased expression of *CIITA* in RSTR monocytes following M. tuberculosis infection is of particular interest in that it may result in reduced MHC-II antigen presentation and, consequently, limit downstream antigen-specific IFN-γ^+^ CD4 T cell responses that fundamentally distinguish the RSTR and LTBI phenotypes. *CIITA* also regulates transcription of other genes that may regulate RSTR pathways, including *BTN2A2*, which also was suppressed following M. tuberculosis infection among RSTRs (Fig. S5B). Interestingly, deficiency of *BTN2A2* in antigen-presenting cells is linked to increased CD4/8 T cell proliferation and decreased frequency of T regulatory cells (37, 38), suggesting relative *BTN2A2* deficiency in RSTR myeloid cells could also enhance T cell functions. Taken together, we identified several top DEGs, including *IFNG*, that are expected based on LTBI phenotype classification, but the relative expression patterns of *CIITA* and other DEGs suggest additional pathways that contribute to the RSTR phenotype.

Our study has several limitations. We did not find any individual genes with significant differential expression in both Uganda and South Africa, which likely reflects either the smaller cohort of gold miners or heterogeneity of these cohorts. Accordingly, transcriptomic analyses from additional RSTR cohorts are needed to validate our findings. Enrichment of the *TNFα* hallmark genes and other gene sets in both Uganda and South Africa cohorts suggests common RSTR mechanisms despite their significant heterogeneity; however, narrowing subsequent analyses to shared genes that drive these enrichments may be required to uncover RSTR mechanisms or to identify molecular targets for host-directed therapies. As discussed previously, the degree to which impurities in our monocyte cultures influenced our differential gene expression due to lymphocyte cytokine paracrine signaling (e.g., *IFNG* and *IL2*) is unknown, but must be considered for genes downstream of these pathways. Another limitation is that resistance to TST/IGRA reactivity is an imperfect surrogate for resistance to infection that cannot be defined microbiologically, and whether RSTR subjects have lower risk of TB progression following exposure is unknown. Finally, to accommodate limitations in participant cell availability, we used a single laboratory M. tuberculosis strain (H37Rv) and chose a single intermediate RNA time point (6 h postinfection) to compare transcriptional profiles from readily accessible peripheral blood. It is likely additional phenotypes would be uncovered according to M. tuberculosis strain and at other time points. Furthermore, similar studies in alveolar macrophages isolated from these cohorts (39), which are the earliest targets for M. tuberculosis infection *in vivo*, may yield different results.

In summary, our study highlights genes and gene pathways that distinguish RSTR and LTBI monocyte responses to M. tuberculosis. These pathways suggest differences in inflammatory responses that may play important roles in mediating resistance to M. tuberculosis infection *in vivo*. Further studies are required to evaluate whether these responses mediate the early clearance of M. tuberculosis from infected myeloid cells or, alternatively, modulate antigen presentation or otherwise impact cell-mediated protective immunity. Elucidating these RSTR pathways may identify targets that augment antimicrobial therapy and vaccine design or may inform diagnostics that better identify M. tuberculosis-exposed individuals to help curb the epidemic.

## MATERIALS AND METHODS

### Subject recruitment.

Recruitment of HIV-negative Uganda household contacts was previously described (8). HIV-negative gold miner participants from South Africa who had worked in the industry for ≥15 years were recruited as part of the Highly Exposed TB Uninfected (HETU) study and underwent baseline TST and IGRA testing; the majority were followed for 12 months for repeat TST/IGRA (10, 40). Informed consent for participation was obtained from each subject at the time of enrollment as detailed in each study protocol that was approved by the respective institutional review boards. RSTR and LTBI phenotype classifications were reported previously with details available in Text S1 in the supplemental material (13).

10.1128/msphere.00159-22.1 TEXT S1 Supplemental methods. Download Text S1, DOCX file, 0.05 MB. Copyright © 2022 Simmons et al. 2022 Simmons et al. https://creativecommons.org/licenses/by/4.0/ This content is distributed under the terms of the Creative Commons Attribution 4.0 International license.

### Culture of M. tuberculosis and monocyte infections.

Log-phase cultures of virulent laboratory Mycobacterium tuberculosis strain H37Rv (a gift from David Sherman) were cultured in supplemented 7H9 medium (Middlebrook) and stored in aliquots at −80°C until monocyte infections to avoid heterogeneity between batches (supplemental material). Monocytes were isolated from cryopreserved peripheral blood mononuclear cells (PBMCs) from each participant and cultured in monocyte colony-stimulated factor (M-CSF) to establish adherence as previously described (13). To minimize artificial homogenization of transcriptional responses due to *ex vivo* culture conditions, experiments were completed within 48 h of PBMC thaw and M-CSF treatment. In a biosafety level 3 (BSL3) laboratory, monocytes were stimulated either with H37Rv (multiplicity of infection [MOI], 1.0) or medium, and RNA was isolated in TRIzol (Invitrogen) after 6 h. These samples were obtained in singlet for donors from South Africa, whereas for Uganda subjects, two independent experiments (Exp A and B in Fig. 1) with partially overlapping subjects were each performed in singlet.

### RNA sequencing and data processing.

Preparation of cDNA libraries, RNA sequencing and alignments using STAR2.6.0a (41), filtering, and analysis were performed in R v4.0.2 (42, 43) (supplemental material). ComBat-seq (44) was used to correct batch effects for Uganda Exp A and B, while controlling for stimulation, phenotype, sex, and age. Counts were converted to log_2_ counts per million using voom (45).

### Differential gene expression, gene set enrichment analyses, and STRING network analysis.

We previously analyzed transcriptomes from unstimulated monocytes from South Africa and a subset of unstimulated Uganda monocytes (Exp A in Fig. 1) (13). To instead identify expression patterns that distinguish RSTR and LTBI phenotypes upon M. tuberculosis stimulation, we selected an expression model that incorporated an interaction term in addition to the main effects: Expression ~ phenotype + stimulation + phenotype:stimulation + covariates, with patient and genetic kinship (when available) included as random effects using R packages coxme (46) or lme4 (47) when kinship data were not available. We adjusted for age, sex, and sequencing batch (Uganda) or age alone for the South Africa samples, which were sequenced simultaneously and given the gold miner participants were all male. Our model sigma and outputs were stable with and without these adjustments (data not shown). Differentially expressed genes (DEGs) in the interaction term (FDR, <0.2) were then assessed using pairwise contrasts of the four phenotype:stimulation groups (e.g., contrasting phenotypes for each stimulation condition or contrasting stimulations for each phenotype) corrected for the same covariates and random effects as the interaction model (supplemental material). To understand biologic connectivity between each of the 260 Uganda DEGs, we used STRING v11 network analysis (48) followed by enrichment analysis using topGO (49). Gene set enrichment analysis (GSEA) was performed using the Molecular Signatures Database (MSigDB v7.2) (16) Hallmark and Gene Ontology (GO) collections. Fast gene set enrichment analysis (FGSEA) (14) was used to compare fold changes of all genes in phenotype:stimulation pairwise contrasts.

### Data availability.

Access to raw transcriptomic data is available through the NCBI database of Genotypes and Phenotypes (dbGaP) Data Browser (https://www.ncbi.nlm.nih.gov/gap/) under accession no. phs002445.v1.p1 (for Uganda) and phs002746.v1.p1 (for South Africa), but first must be approved by data access committees (DACs) for each study site (supplemental material). All R code is available at https://github.com/hawn-lab/RSTR_RNAseq_Mtb_public.

## References

[1] Lu LL, Smith MT, Yu KKQ, Luedemann C, Suscovich TJ, Grace PS, Cain A, Yu WH, McKitrick TR, Lauffenburger D, Cummings RD, Mayanja-Kizza H, Hawn TR, Boom WH, Stein CM, Fortune SM, Seshadri C, Alter G. 2019. IFN-gamma-independent immune markers of Mycobacterium tuberculosis exposure. Nat Med 25:977–987. doi:10.1038/s41591-019-0441-3. 31110348 PMC6559862

[2] Verrall AJ, Schneider M, Alisjahbana B, Apriani L, van Laarhoven A, Koeken V, van Dorp S, Diadani E, Utama F, Hannaway RF, Indrati A, Netea MG, Sharples K, Hill PC, Ussher JE, van Crevel R. 2020. Early clearance of Mycobacterium tuberculosis is associated with increased innate immune responses. J Infect Dis 221:1342–1350. doi:10.1093/infdis/jiz147. 30958547

[3] Weiner J, Domaszewska T, Donkor S, Kaufmann SHE, Hill PC, Sutherland JS. 2020. Changes in transcript, metabolite, and antibody reactivity during the early protective immune response in humans to Mycobacterium tuberculosis infection. Clin Infect Dis 71:30–40. doi:10.1093/cid/ciz785. 31412355 PMC7312225

[4] Medawar L, Tukiman HM, Mbayo G, Donkor S, Owolabi O, Sutherland JS. 2019. Analysis of cellular and soluble profiles in QuantiFERON nonconverters, converters, and reverters in the Gambia. Immun Inflamm Dis 7:260–270. doi:10.1002/iid3.269. 31430056 PMC6842814

[5] Boom WH, Schaible UE, Achkar JM. 2021. The knowns and unknowns of latent Mycobacterium tuberculosis infection. J Clin Invest 131:e136222. doi:10.1172/JCI136222. PMC7843221 33529162

[6] Simmons JD, Stein CM, Seshadri C, Campo M, Alter G, Fortune S, Schurr E, Wallis RS, Churchyard G, Mayanja-Kizza H, Boom WH, Hawn TR. 2018. Immunological mechanisms of human resistance to persistent Mycobacterium tuberculosis infection. Nat Rev Immunol 18:575–589. doi:10.1038/s41577-018-0025-3. 29895826 PMC6278832

[7] Gutierrez J, Kroon EE, Moller M, Stein CM. 2021. Phenotype definition for “resisters” to Mycobacterium tuberculosis infection in the literature—a review and recommendations. Front Immunol 12:619988. doi:10.3389/fimmu.2021.619988. 33717116 PMC7946835

[8] Stein CM, Nsereko M, Malone LL, Okware B, Kisingo H, Nalukwago S, Chervenak K, Mayanja-Kizza H, Hawn TR, Boom WH. 2019. Long-term stability of resistance to latent Mycobacterium tuberculosis infection in highly exposed tuberculosis household contacts in Kampala, Uganda. Clin Infect Dis 68:1705–1712. doi:10.1093/cid/ciy751. 30165605 PMC6495009

[9] Stein CM, Zalwango S, Malone LL, Thiel B, Mupere E, Nsereko M, Okware B, Kisingo H, Lancioni CL, Bark CM, Whalen CC, Joloba ML, Boom WH, Mayanja-Kizza H. 2018. Resistance and susceptibility to Mycobacterium tuberculosis infection and disease in tuberculosis households in Kampala, Uganda. Am J Epidemiol 187:1477–1489. doi:10.1093/aje/kwx380. 29304247 PMC6031055

[10] Chihota V, Ntshiqa T, Maenetje P, Mansukhani R, Velen K, Hawn TR, Wallis RS, Grant AD, Churchyard GJ, Fielding KL. 2022. Resistance to Mycobacterium tuberculosis infection among highly TB exposed South African gold miners. PLoS One 17:e0265036. doi:10.1371/journal.pone.0265036. 35302992 PMC8932619

[11] Verrall AJ, Netea MG, Alisjahbana B, Hill PC, van Crevel R. 2014. Early clearance of Mycobacterium tuberculosis: a new frontier in prevention. Immunology 141:506–513. doi:10.1111/imm.12223. 24754048 PMC3956425

[12] Vorkas CK, Wipperman MF, Li K, Bean J, Bhattarai SK, Adamow M, Wong P, Aubé J, Juste MAJ, Bucci V, Fitzgerald DW, Glickman MS. 2018. Mucosal-associated invariant and gammadelta T cell subsets respond to initial Mycobacterium tuberculosis infection. JCI Insight 3:e121899. doi:10.1172/jci.insight.121899. PMC6237486 30282828

[13] Simmons JD, Van PT, Stein CM, Chihota V, Ntshiqa T, Maenetje P, Peterson GJ, Reynolds A, Benchek P, Velen K, Fielding KL, Grant AD, Graustein AD, Nguyen FK, Seshadri C, Gottardo R, Mayanja-Kizza H, Wallis RS, Churchyard G, Boom WH, Hawn TR. 2021. Monocyte metabolic transcriptional programs associate with resistance to tuberculin skin test/interferon-gamma release assay conversion. J Clin Invest 131:e140073. doi:10.1172/JCI140073. PMC8279582 34111032

[14] Korotkevich G, Sukhov V, Budin N, Shpak B, Artyomov MN, Sergushichev A. 2021. Fast gene set enrichment analysis. bioRxiv 10.1101/060012.

[15] Subramanian A, Tamayo P, Mootha VK, Mukherjee S, Ebert BL, Gillette MA, Paulovich A, Pomeroy SL, Golub TR, Lander ES, Mesirov JP. 2005. Gene set enrichment analysis: a knowledge-based approach for interpreting genome-wide expression profiles. Proc Natl Acad Sci USA 102:15545–15550. doi:10.1073/pnas.0506580102. 16199517 PMC1239896

[16] Liberzon A, Birger C, Thorvaldsdottir H, Ghandi M, Mesirov JP, Tamayo P. 2015. The Molecular Signatures Database (MSigDB) hallmark gene set collection. Cell Syst 1:417–425. doi:10.1016/j.cels.2015.12.004. 26771021 PMC4707969

[17] Gleeson LE, Sheedy FJ, Palsson-McDermott EM, Triglia D, O'Leary SM, O'Sullivan MP, O'Neill LA, Keane J. 2016. Cutting edge: Mycobacterium tuberculosis induces aerobic glycolysis in human alveolar macrophages that is required for control of intracellular bacillary replication. J Immunol 196:2444–2449. doi:10.4049/jimmunol.1501612. 26873991

[18] Hackett EE, Charles-Messance H, O'Leary SM, Gleeson LE, Munoz-Wolf N, Case S, Wedderburn A, Johnston DGW, Williams MA, Smyth A, Ouimet M, Moore KJ, Lavelle EC, Corr SC, Gordon SV, Keane J, Sheedy FJ. 2020. Mycobacterium tuberculosis limits host glycolysis and IL-1beta by restriction of PFK-M via microRNA-21. Cell Rep 30:124–136.e4. doi:10.1016/j.celrep.2019.12.015. 31914380 PMC7764301

[19] Cobat A, Gallant CJ, Simkin L, Black GF, Stanley K, Hughes J, Doherty TM, Hanekom WA, Eley B, Jais JP, Boland-Auge A, van Helden P, Casanova JL, Abel L, Hoal EG, Schurr E, Alcais A. 2009. Two loci control tuberculin skin test reactivity in an area hyperendemic for tuberculosis. J Exp Med 206:2583–2591. doi:10.1084/jem.20090892. 19901083 PMC2806605

[20] Cobat A, Poirier C, Hoal E, Boland-Auge A, de La Rocque F, Corrard F, Grange G, Migaud M, Bustamante J, Boisson-Dupuis S, Casanova JL, Schurr E, Alcais A, Delacourt C, Abel L. 2015. Tuberculin skin test negativity is under tight genetic control of chromosomal region 11p14-15 in settings with different tuberculosis endemicities. J Infect Dis 211:317–321. doi:10.1093/infdis/jiu446. 25143445 PMC4279780

[21] Igo RP, Jr, Hall NB, Malone LL, Hall JB, Truitt B, Qiu F, Tao L, Mupere E, Schnell A, Hawn TR, Bush WS, Joloba M, Boom WH, Stein CM. 2019. Fine-mapping analysis of a chromosome 2 region linked to resistance to Mycobacterium tuberculosis infection in Uganda reveals potential regulatory variants. Genes Immun 20:473–483. doi:10.1038/s41435-018-0040-1. 30100616 PMC6374218

[22] Stein CM, Zalwango S, Malone LL, Won S, Mayanja-Kizza H, Mugerwa RD, Leontiev DV, Thompson CL, Cartier KC, Elston RC, Iyengar SK, Boom WH, Whalen CC. 2008. Genome scan of M. tuberculosis infection and disease in Ugandans. PLoS One 3:e4094. doi:10.1371/journal.pone.0004094. 19116662 PMC2605555

[23] Hall NB, Igo RP, Jr, Malone LL, Truitt B, Schnell A, Tao L, Okware B, Nsereko M, Chervenak K, Lancioni C, Hawn TR, Mayanja-Kizza H, Joloba ML, Boom WH, Stein CM, Tuberculosis Research Unit. 2015. Polymorphisms in TICAM2 and IL1B are associated with TB. Genes Immun 16:127–133. doi:10.1038/gene.2014.77. 25521228 PMC4352113

[24] Flynn JL, Goldstein MM, Chan J, Triebold KJ, Pfeffer K, Lowenstein CJ, Schreiber R, Mak TW, Bloom BR. 1995. Tumor necrosis factor-alpha is required in the protective immune response against Mycobacterium tuberculosis in mice. Immunity 2:561–572. doi:10.1016/1074-7613(95)90001-2. 7540941

[25] Keane J, Gershon S, Wise RP, Mirabile-Levens E, Kasznica J, Schwieterman WD, Siegel JN, Braun MM. 2001. Tuberculosis associated with infliximab, a tumor necrosis factor alpha-neutralizing agent. N Engl J Med 345:1098–1104. doi:10.1056/NEJMoa011110. 11596589

[26] Mohan AK, Cote TR, Block JA, Manadan AM, Siegel JN, Braun MM. 2004. Tuberculosis following the use of etanercept, a tumor necrosis factor inhibitor. Clin Infect Dis 39:295–299. doi:10.1086/421494. 15306993

[27] Gomez-Reino JJ, Carmona L, Valverde VR, Mola EM, Montero MD, BIOBADASER Group. 2003. Treatment of rheumatoid arthritis with tumor necrosis factor inhibitors may predispose to significant increase in tuberculosis risk: a multicenter active-surveillance report. Arthritis Rheum 48:2122–2127. doi:10.1002/art.11137. 12905464

[28] Cobat A, Hoal EG, Gallant CJ, Simkin L, Black GF, Stanley K, Jais JP, Yu TH, Boland-Auge A, Grange G, Delacourt C, van Helden P, Casanova JL, Abel L, Alcais A, Schurr E. 2013. Identification of a major locus, TNF1, that controls BCG-triggered tumor necrosis factor production by leukocytes in an area hyperendemic for tuberculosis. Clin Infect Dis 57:963–970. doi:10.1093/cid/cit438. 23800941 PMC3765013

[29] Jeffrey KL, Brummer T, Rolph MS, Liu SM, Callejas NA, Grumont RJ, Gillieron C, Mackay F, Grey S, Camps M, Rommel C, Gerondakis SD, Mackay CR. 2006. Positive regulation of immune cell function and inflammatory responses by phosphatase PAC-1. Nat Immunol 7:274–283. doi:10.1038/ni1310. 16474395

[30] Lu D, Liu L, Ji X, Gao Y, Chen X, Liu Y, Liu Y, Zhao X, Li Y, Li Y, Jin Y, Zhang Y, McNutt MA, Yin Y. 2015. The phosphatase DUSP2 controls the activity of the transcription activator STAT3 and regulates TH17 differentiation. Nat Immunol 16:1263–1273. doi:10.1038/ni.3278. 26479789

[31] Lee J, Boyce S, Powers J, Baer C, Sassetti CM, Behar SM. 2020. CD11cHi monocyte-derived macrophages are a major cellular compartment infected by Mycobacterium tuberculosis. PLoS Pathog 16:e1008621. doi:10.1371/journal.ppat.1008621. 32544188 PMC7319360

[32] Stavrum R, Valvatne H, Stavrum AK, Riley LW, Ulvestad E, Jonassen I, Doherty TM, Grewal HM. 2012. Mycobacterium tuberculosis Mce1 protein complex initiates rapid induction of transcription of genes involved in substrate trafficking. Genes Immun 13:496–502. doi:10.1038/gene.2012.24. 22695749

[33] Pei L, Castrillo A, Chen M, Hoffmann A, Tontonoz P. 2005. Induction of NR4A orphan nuclear receptor expression in macrophages in response to inflammatory stimuli. J Biol Chem 280:29256–29262. doi:10.1074/jbc.M502606200. 15964844

[34] Mahajan S, Saini A, Chandra V, Nanduri R, Kalra R, Bhagyaraj E, Khatri N, Gupta P. 2015. Nuclear receptor Nr4a2 promotes alternative polarization of macrophages and confers protection in sepsis. J Biol Chem 290:18304–18314. doi:10.1074/jbc.M115.638064. 25953901 PMC4513091

[35] Ishizawa M, Kagechika H, Makishima M. 2012. NR4A nuclear receptors mediate carnitine palmitoyltransferase 1A gene expression by the rexinoid HX600. Biochem Biophys Res Commun 418:780–785. doi:10.1016/j.bbrc.2012.01.102. 22310716

[36] Schurz H, Kinnear CJ, Gignoux C, Wojcik G, van Helden PD, Tromp G, Henn B, Hoal EG, Moller M. 2018. A sex-stratified genome-wide association study of tuberculosis using a multi-ethnic genotyping array. Front Genet 9:678. doi:10.3389/fgene.2018.00678. 30713548 PMC6346682

[37] Sarter K, Leimgruber E, Gobet F, Agrawal V, Dunand-Sauthier I, Barras E, Mastelic-Gavillet B, Kamath A, Fontannaz P, Guéry L, Duraes F.dV, Lippens C, Ravn U, Santiago-Raber M-L, Magistrelli G, Fischer N, Siegrist C-A, Hugues S, Reith W. 2016. Btn2a2, a T cell immunomodulatory molecule coregulated with MHC class II genes. J Exp Med 213:177–187. doi:10.1084/jem.20150435. 26809444 PMC4749920

[38] Ammann JU, Cooke A, Trowsdale J. 2013. Butyrophilin Btn2a2 inhibits TCR activation and phosphatidylinositol 3-kinase/Akt pathway signaling and induces Foxp3 expression in T lymphocytes. J Immunol 190:5030–5036. doi:10.4049/jimmunol.1203325. 23589618 PMC3736090

[39] Thiel BA, Worodria W, Nalukwago S, Nsereko M, Sanyu I, Rejani L, Zawedde J, Canaday DH, Stein CM, Chervenak KA, Malone LL, Kiyemba R, Silver RF, Johnson JL, Mayanja-Kizza H, Boom WH. 2021. Immune cells in bronchoalveolar lavage fluid of Ugandan adults who resist versus those who develop latent Mycobacterium tuberculosis infection. PLoS One 16:e0249477. doi:10.1371/journal.pone.0249477. 33836031 PMC8034721

[40] Ntshiqa T, Chihota V, Mansukhani R, Nhlangulela L, Velen K, Charalambous S, Maenetje P, Hawn TR, Wallis R, Grant AD, Fielding K, Churchyard G. 2021. Comparing the performance of QuantiFERON-TB Gold Plus with QuantiFERON-TB Gold in-tube among highly TB exposed gold miners in South Africa. Gates Open Res 5:66. doi:10.12688/gatesopenres.13191.2. PMC10407057 37560544

[41] Dobin A, Davis CA, Schlesinger F, Drenkow J, Zaleski C, Jha S, Batut P, Chaisson M, Gingeras TR. 2013. STAR: ultrafast universal RNA-seq aligner. Bioinformatics 29:15–21. doi:10.1093/bioinformatics/bts635. 23104886 PMC3530905

[42] R Core Team. 2020. R: a language and environment for statistical computing, v4.0.2. R Foundation for Statistical Computing, Vienna, Austria. https://www.R-project.org/.

[43] Wickham H, Averick M, Bryan J, Chang W, McGowan LDA, François R, Grolemund G, Hayes A, Henry L, Hester J, Kuhn M, Pedersen TL, Miller E, Bache SM, Müller K, Ooms J, Robinson D, Seidel DP, Spinu V, Takahashi K, Vaughan D, Wilke C, Woo K, Yutani H. 2019. Welcome to the Tidyverse. Joss 4:1686–1686. doi:10.21105/joss.01686.

[44] Zhang Y, Parmigiani G, Johnson WE. 2020. ComBat-seq: batch effect adjustment for RNA-seq count data. NAR Genom Bioinform 2:lqaa078. doi:10.1093/nargab/lqaa078. 33015620 PMC7518324

[45] Law CW, Chen Y, Shi W, Smyth GK. 2014. voom: precision weights unlock linear model analysis tools for RNA-seq read counts. Genome Biol 15:R29. doi:10.1186/gb-2014-15-2-r29. 24485249 PMC4053721

[46] Therneau TM. 2020. coxme: Mixed Effects Cox Models, v2.2–16. CRAN https://cran.r-project.org/package=coxme.

[47] Bates D, Mächler M, Bolker B, Walker S. 2015. Fitting linear mixed-effects models using lme4. J Stat Software 67:48.

[48] Szklarczyk D, Gable AL, Lyon D, Junge A, Wyder S, Huerta-Cepas J, Simonovic M, Doncheva NT, Morris JH, Bork P, Jensen LJ, Mering CV. 2019. STRING v11: protein-protein association networks with increased coverage, supporting functional discovery in genome-wide experimental datasets. Nucleic Acids Res 47:D607–D613. doi:10.1093/nar/gky1131. 30476243 PMC6323986

[49] Alexa A, Rahnenfuhrer J. 2021. topGO: enrichment analysis for Gene Ontology. https://bioconductor.org/packages/release/bioc/html/topGO.html.

