# Supplementary material for: Monocyte Transcriptional Responses to Mycobacterium tuberculosis Associate with Resistance to Tuberculin Skin Test and Interferon Gamma Release Assay Conversion
Source: mSphere. 2022 Jun 13;7(3):e00159-22. doi: 10.1128/msphere.00159-22 (PMC9241521; doi:10.1128/msphere.00159-22)
Supplement: TABLE S1 [file msphere.00159-22-s0007.docx]

**Supplemental Table 1: Comparison of demographic and epidemiologic characteristics of Ugandan subjects**

|  | **RSTR** | **LTBI** | ***P* value *^A^*** |
| --- | --- | --- | --- |
| N | 49 | 52 |  |
| Age |  |  |  |
| Age enroll, median (IQR)  Age re-trace, median (IQR) | 13 (9)  21 (9) | 13 (8)  22 (7.5) | 0.55  0.44 |
| Sex, % male (n/N) | 55.1(27/49) | 57.7 (30/52) | 0.95 |
| BMI, median (IQR)** | 21.0 (5.33) | 22.4 (3.97) | 0.12 |
| % BCG scar (n/N) | 77.5 (31/40) | 74.4 (32/43) | 0.95 |
| Exposure score, mean (SD) *^B^* | 6.18 (1.15) | 6.42 (1.18) | 0.19 |
| Relatedness within phenotype *^C^* |  |  |  |
| Mean 3^o^ & closer relations per person (SD) | 0.78 (0.90) | 0.62 (0.84) | 0.28 |
| Mean 1^o^ & closer relations per person (SD) | 0.67 (0.60) | 0.50 (0.78) | 0.57 |

*^A^* Statistical comparisons were made using Pearson Chi-square (categorical variables) or two-sample Wilcoxon rank-sum (Mann-Whitney) tests (continuous variables).

*^B^*Exposure scores were calculated using the adult or pediatric risk score according to patient age at initial enrollment (<15 years with the Pediatric risk score, ≥ 15 years with the Adult Exposure Risk Score) as previously described.

*^C^* Relatedness of subjects within each phenotype were compared by averaging the number of 1^st^ degree relationships and 3^rd^ degree-or-closer relationships. BMI, body mass index; IQR, interquartile range; n/N, (number subjects counted/number of subjects with available data); SD, standard deviation.

** Missing BMI in 5 subjects (2 LTBI and 3 RSTR).
