# Supplementary material for: Monocyte Transcriptional Responses to Mycobacterium tuberculosis Associate with Resistance to Tuberculin Skin Test and Interferon Gamma Release Assay Conversion
Source: mSphere. 2022 Jun 13;7(3):e00159-22. doi: 10.1128/msphere.00159-22 (PMC9241521; doi:10.1128/msphere.00159-22)
Supplement: TABLE S2 [file msphere.00159-22-s0008.docx]

**Supplemental Table 2: Comparison of demographic and epidemiologic characteristics of South African subjects**

|  | **RSTR** | **LTBI** | **p-value *^A^*** |
| --- | --- | --- | --- |
| N | 26 | 29 |  |
| Age at enrollment, median (IQR) | 50 (8.5) | 49 (5.0) | 0.99 |
| Sex (% male) | 100 | 100 |  |
| BMI, median (IQR) | 27.6 (4.83) | 31.0 (5) | 0.09 |
| Yrs worked underground, median (IQR) | 21.5 (9.5) | 26.0 (11.0) | 0.75 |
| Ancestry |  |  | 0.011 |
| % Black/African (n/N) | 73.1 (19/26) | 100.0 (29/29) |  |
| % White/European (n/N)  % Other (n/N) | 23.1 (6/26)  3.85 (1/26) | 0  0 |  |
| % BCG Scar (n/N) | 68 (17/25) | 69 (20/29) | 0.94 |

*^A^* Statistical comparisons were made using Pearson Chi-square or Fisher’s exact test (categorical variables) or two-sample Wilcoxon rank-sum (Mann-Whitney) tests. BMI, body mass index; IQR, interquartile range; n/N, (number subjects counted/number of subjects with available data).
