# Supplementary figures and images for: Monocyte Transcriptional Responses to Mycobacterium tuberculosis Associate with Resistance to Tuberculin Skin Test and Interferon Gamma Release Assay Conversion
Source: mSphere. 2022 Jun 13;7(3):e00159-22. doi: 10.1128/msphere.00159-22 (PMC9241521; doi:10.1128/msphere.00159-22)

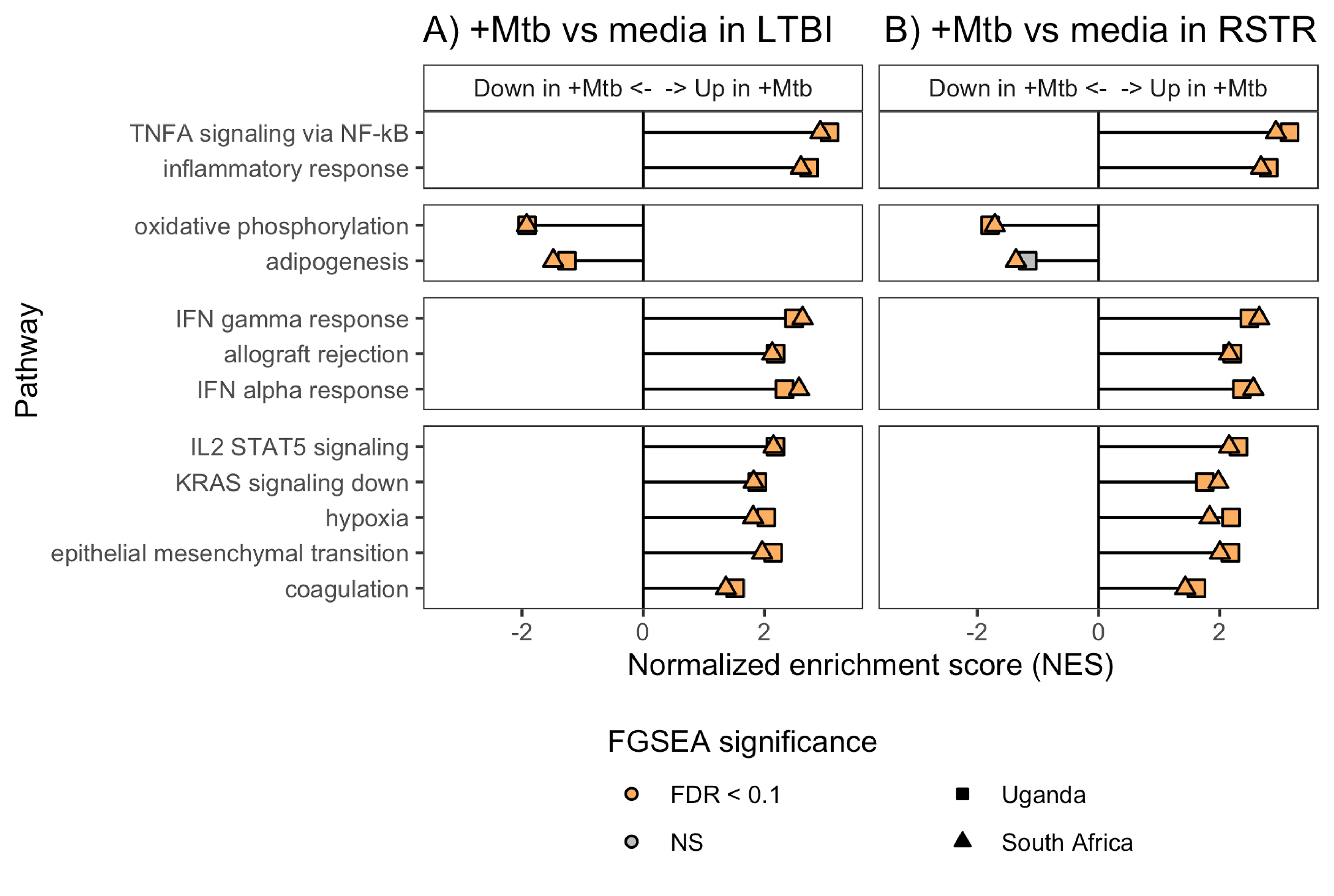

Supplement: FIG S1 [file msphere.00159-22-s0002.tif]

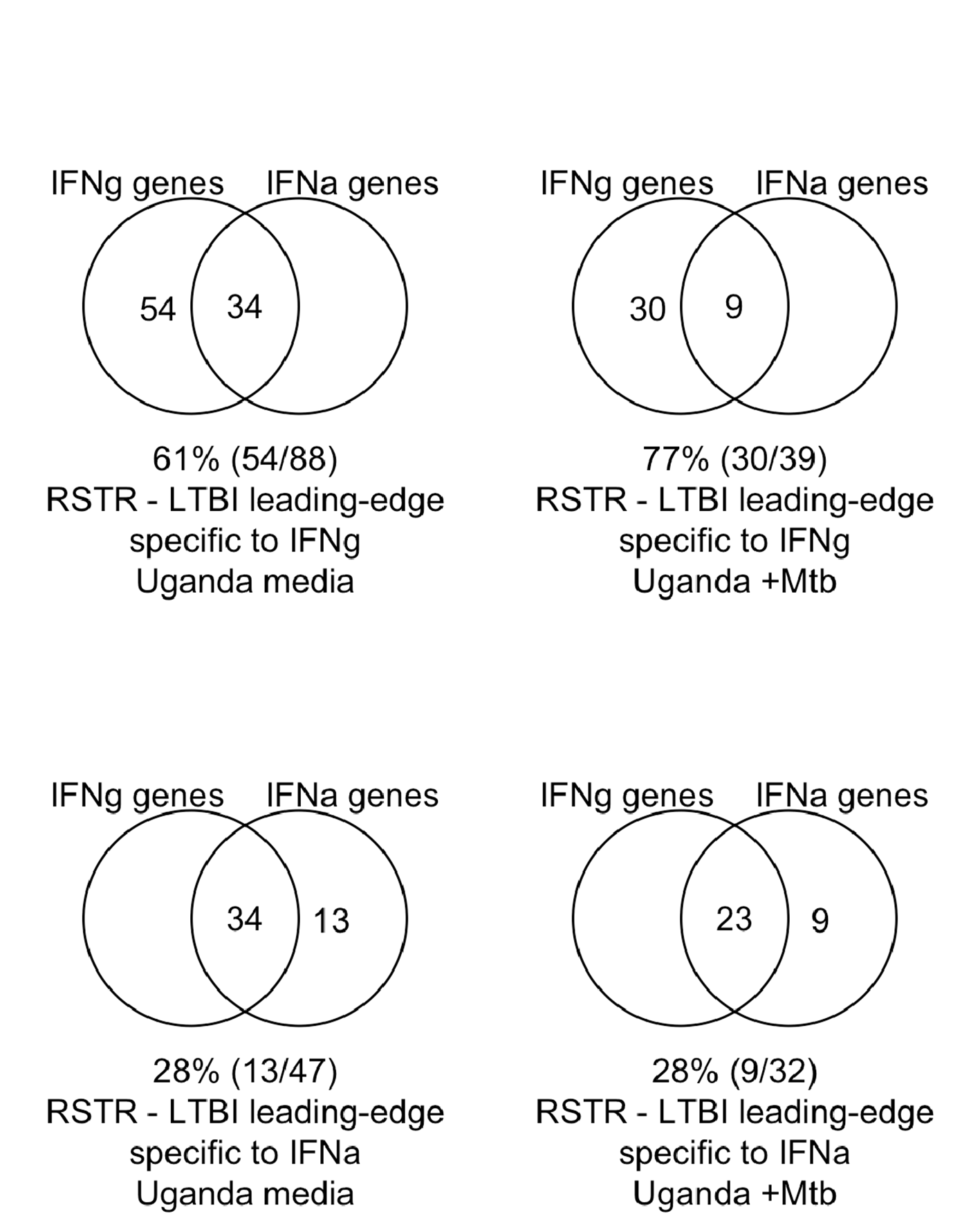

Supplement: FIG S2 [file msphere.00159-22-s0003.tif]

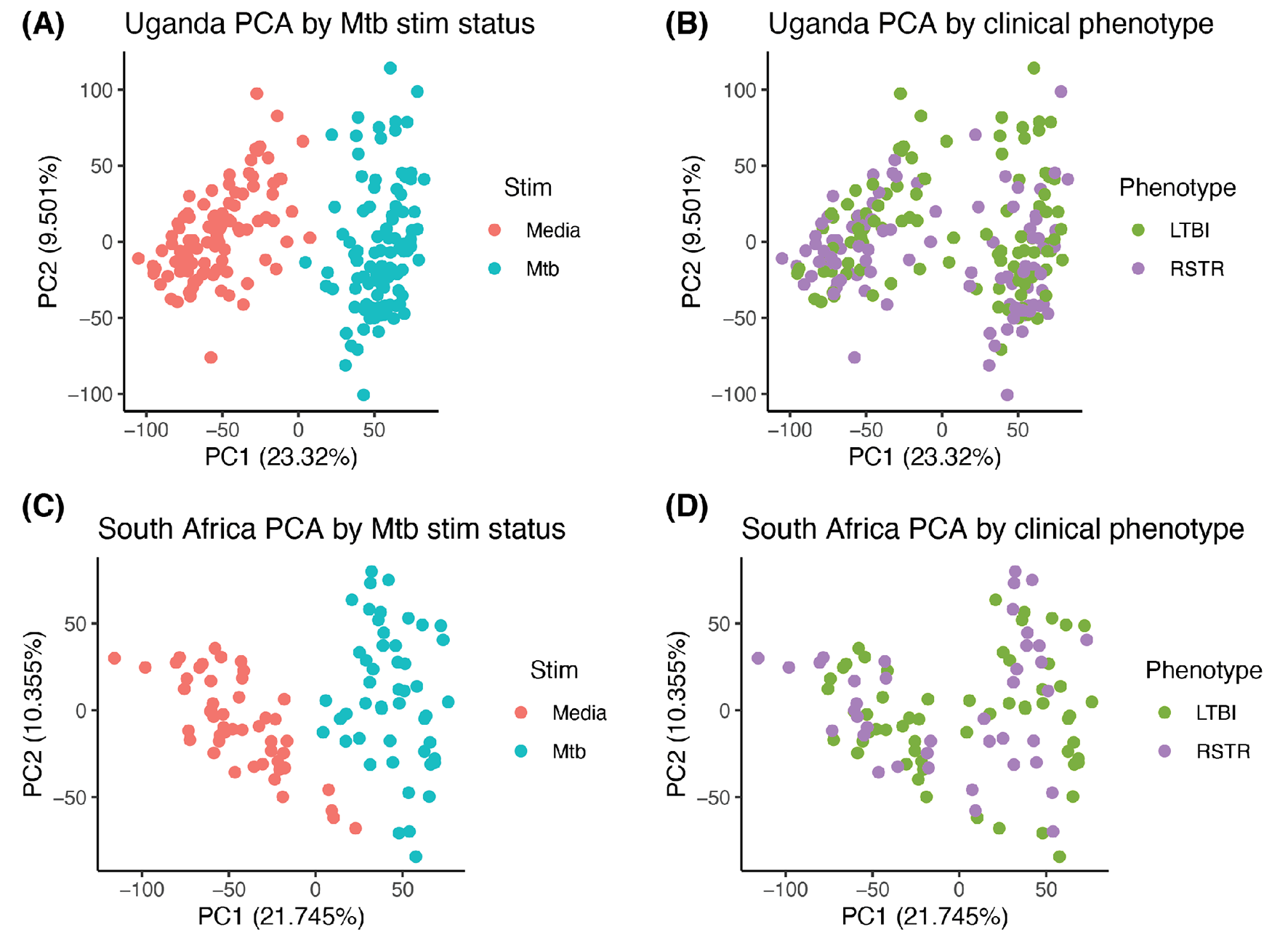

Supplement: FIG S3 [file msphere.00159-22-s0004.tif]

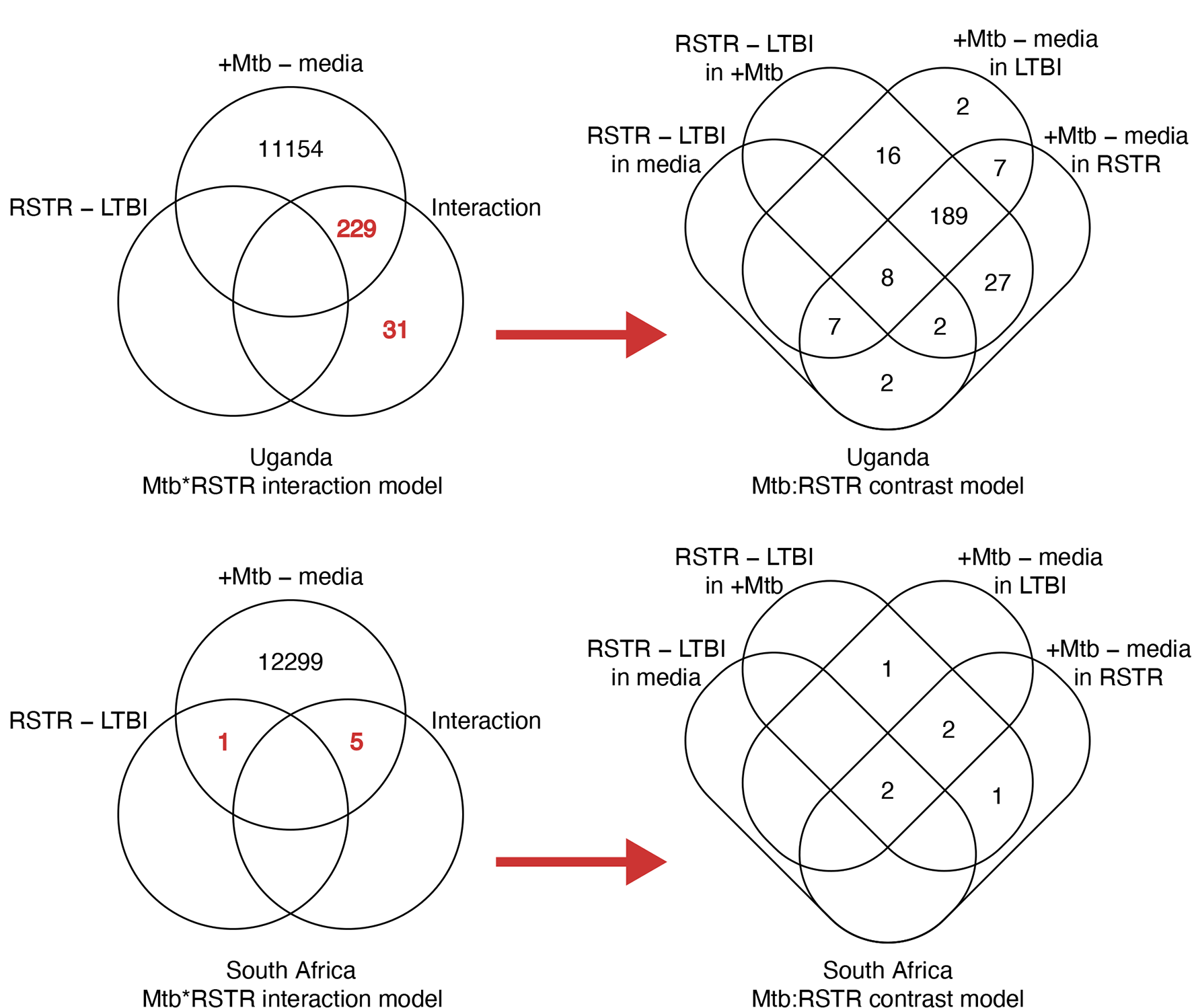

Supplement: FIG S4 [file msphere.00159-22-s0005.tif]

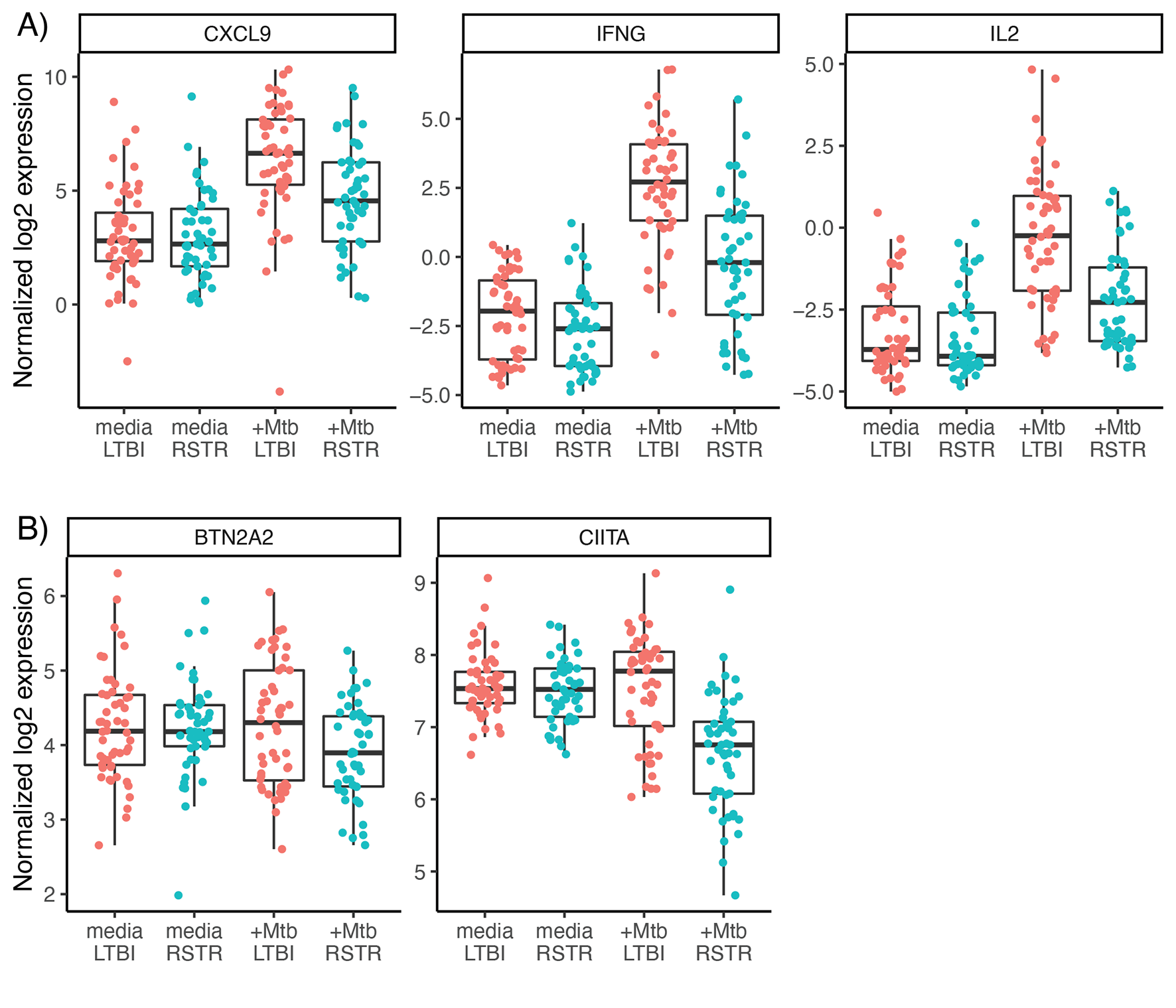

Supplement: FIG S5 [file msphere.00159-22-s0006.tif]
