# Supplementary material for: Monocyte Transcriptional Responses to Mycobacterium tuberculosis Associate with Resistance to Tuberculin Skin Test and Interferon Gamma Release Assay Conversion
Source: mSphere. 2022 Jun 13;7(3):e00159-22. doi: 10.1128/msphere.00159-22 (PMC9241521; doi:10.1128/msphere.00159-22)
Supplement: TABLE S4 [file msphere.00159-22-s0010.docx]

**Supplemental Table 4: Uganda Mtb*RSTR DEG polymorphisms associate with clinical phenotype**

|  |  |  | **Genotype frequency** | | |  |  |
| --- | --- | --- | --- | --- | --- | --- | --- |
| **Gene** | **Total SNPs** | **SNP** | **Genotype** | **RSTR (case)** | **LTBI (control)** | **OR^A^**  **(95% CI)** | **P value** |
| EPB41L3 | 105 | rs1719945 | GG | 0.42 | 0.59 | 1.95  (1.23, 3.09) | 4.75E-03 |
|  |  |  | GA | 0.46 | 0.36 |  |  |
|  |  |  | AA | 0.12 | 0.05 |  |  |
| EPB41L3 | 105 | rs183161014 | AA | 0.66 | 0.81 | 2.22  (1.33, 3.68) | 2.16-03 |
|  |  |  | AG | 0.22 | 0.16 |  |  |
|  |  |  | GG | 0.12 | 0.03 |  |  |
| ZNF184 | 5 | rs1883216 | CC | 0.19 | 0.32 | 1.88  (1.23, 2.86) | 3.31E-03 |
|  |  |  | CA | 0.51 | 0.53 |  |  |
|  |  |  | AA | 0.30 | 0.15 |  |  |
| AKT3 | 42 | rs12144559 | CC | 0.96 | 0.80 | 0.30  (0.14, 0.65) | 2.27E-03 |
|  |  |  | CA | 0.04 | 0.18 |  |  |
|  |  |  | AA | 0.00 | 0.02 |  |  |
| KLHL29 | 122 | rs1530045 | GG | 0.41 | 0.55 | 1.92  (1.22, 3.03) | 4.77E-03 |
|  |  |  | GA | 0.42 | 0.40 |  |  |
|  |  |  | AA | 0.16 | 0.05 |  |  |
| CUX1 | 99 | rs12672026 | GG | 0.20 | 0.40 | 1.95  (1.29, 2.95) | 1.52E-03 |
|  |  |  | GA | 0.53 | 0.46 |  |  |
|  |  |  | AA | 0.27 | 0.14 |  |  |
| ST3GAL1 | 82 | rs56121321 | AA | 0.23 | 0.41 | 1.88  (1.23, 2.86) | 3.33E-03 |
|  |  |  | AG | 0.54 | 0.46 |  |  |
|  |  |  | GG | 0.23 | 0.13 |  |  |
| APOL4 | 23 | rs12170983 | AA | 0.59 | 0.79 | 2.66  (1.45, 4.89) | 1.66E-03 |
|  |  |  | AG | 0.38 | 0.20 |  |  |
|  |  |  | GG | 0.03 | 0.01 |  |  |
| LCP1 | 36 | rs7330090 | GG | 0.55 | 0.73 | 2.18  (1.29, 3.67) | 3.37E-03 |
|  |  |  | GA | 0.36 | 0.25 |  |  |
|  |  |  | AA | 0.08 | 0.02 |  |  |
| TBC1D31 | 30 | rs2385159 | AA | 0.76 | 0.89 | 2.92  (1.39, 6.12) | 4.48E-03 |
|  |  |  | AT | 0.22 | 0.11 |  |  |
|  |  |  | TT | 0.03 | 0.00 |  |  |
| CIITA | 31 | rs6498130 | CC | 0.23 | 0.32 | 1.92  (1.26, 2.91) | 2.20E-03 |
|  |  |  | CA | 0.41 | 0.54 |  |  |
|  |  |  | AA | 0.36 | 0.14 |  |  |

^A^ OR, odds ratio, indicates additive odds for being a case (RSTR) compared to control (LTBI) for each copy of the minor allele inherited (additive model). Note, 5248 SNPs were examined across 260 DEGs.
