# Supplementary material for: Monocyte Transcriptional Responses to Mycobacterium tuberculosis Associate with Resistance to Tuberculin Skin Test and Interferon Gamma Release Assay Conversion
Source: mSphere. 2022 Jun 13;7(3):e00159-22. doi: 10.1128/msphere.00159-22 (PMC9241521; doi:10.1128/msphere.00159-22)
Supplement: TEXT S1 [file msphere.00159-22-s0001.docx]

**Supplementary Material**

**Supplemental Methods**

*Subject recruitment*

HIV-negative household contacts of pulmonary Mtb cases were recruited as part of the Kawempe Community Health Study (Kampala, Uganda) and followed with serial TST testing for two years (1); a subset of participants, which were used in the current study, were re-contacted 8-10 years later for collection of PBMCs, a repeat TST, and the performance of three IGRA tests, as previously described (2). Gold miner participants from South Africa were recruited as part of the Highly Exposed TB Uninfected (HETU) study where inclusion and exclusion criteria are described elsewhere (3, 4). Briefly, these participants worked in the mining industry for ≥15 years, were HIV-negative, and had no prior treatments for tuberculosis. These miners underwent baseline TST and IGRA testing (analysis restricted to use of Quantiferon-Gold In-Tube [QFT-GIT] results to remain consistent with Uganda study), and the majority were followed for 12 months for repeat TST/IGRA. Our phenotype classifications were reported previously (5) where RSTR subjects included Uganda participants meeting the ‘definite’ category (2) who had concordant negative TST/IGRA results at all available collections. In South Africa, RSTR subjects also had concordant negative results from all available TST/IGRA tests, the majority (n = 22/26) of whom had 12-month follow-up testing. LTBI control subjects were defined by concordant, positive TST/IGRA testing on all available tests in both studies.

*Cell culture reagents, mycobacterial strains*

Monocytes were cultured in Roswell Park Memorial Institute 1640 medium containing phenol red, HEPES and L-glutamine (RPMI 1640, Gibco) supplemented with fetal bovine serum (Atlas Biologicals) to a final concentration of 10% (RPMI-10) and with macrophage colony-stimulating factor (MCSF, Peprotech) at 50 ng/mL. The virulent laboratory *Mycobacterium tuberculosis* strain H37Rv (gift of David Sherman) was cultured in 7H9 media (Middlebrook) supplemented with glycerol (Fisher; 4 mL/L), albumin-dextrose-catalase (ADC) supplement (Middlebrook, 100 mL/L) and Tween 80 (Fisher; 0.05% final) and grown to log-phase. Cultures were pelleted at 3,000 x g, washed twice in Sauton’s media, resuspended in Sauton’s media to OD ~1.0 and aliquots were frozen at -80^o^C until monocyte infections. Freshly thawed H37Rv stocks were used to immediately infect monocyte cultures after obtaining the optical density to avoid heterogeneity between batches. The conversion of OD to CFU to achieve the desired multiplicity of infection (MOI) was determined by plating serial dilutions of a freshly frozen stock on 7H10 agar (Middlebrook) for CFU enumeration.

*CD14+ monocyte isolation and Mtb infection*

Cryopreserved PBMCs were shipped from the respective study sites and maintained under liquid nitrogen. In batches of 5-6 donors (balanced by RSTR/LTBI phenotype), PBMCs were thawed (Day = 0) and viable cells, as assessed by Trypan Blue stain, were resuspended in RPMI/10 containing MCSF (50 ng/mL) at 2 million cells per mL and rested overnight in non-TC treated dishes at 37 ^o^C. On day 1, CD14+ monocytes were enriched with negative selection using magnetic beads (Monocyte Isolation Kit II, Miltenyi Biotec) and then plated at 1 million cells per mL RPMI-10 supplemented with MCSF and again incubated at 37 ^o^C. The purity of the enriched CD14+ population was 60-80% as determined by flow cytometry. On day 2, cell cultures were taken into the BSL3 laboratory and stimulated either with H37Rv diluted in Sauton’s media to achieve an estimated MOI 1.0 or an equivalent volume of Sauton’s media alone. After 6 hours, media was aspirated and cells were lysed in Trizol (Invitrogen) and lysates were transferred to cryotubes, removed from BSL3 and stored at -80 ^o^C. RNA was isolated from lysates in batches by chloroform extraction and the application of the aqueous phase with 100% ethanol to miRNeasy micro columns, which were washed and eluted according to the manufacturer instructions (Qiagen). RNA quality was assessed by Agilent TapeStation to ensure RIN ≥ 8.0 and quantification was measured using Nanodrop (Thermo Scientific). These samples were obtained in singlet for donors from South Africa whereas for Uganda subjects, two independent experiments (Exp A and B, Figure 1) with partially overlapping subjects were each performed in singlet.

*RNA sequencing and data processing*

Preparation of cDNA libraries, RNA sequencing and alignments were performed as previously described in detail(5) on the Illumina Hiseq 2500 (high-output mode). A second experiment (Exp B, Figure 1) was performed on monocytes from subjects partially overlapping with the prior experiment and processed equivalently for RNA sequencing on the Novaseq 6000. Sequences were aligned to the GRCH38 reference genome with STAR2.6.0a (6). Counts were assigned to gene exons using RSEM 1.3.0. Further RNA sequencing data filtering and analysis were performed in R v4.0.2 (7, 8). All sequencing libraries achieved sufficient depth with 11 million ± 4 million s.d. reads per library, and no outliers were apparent in principal component analysis (PCA). ComBat-seq (9) was used to correct batch effects for Uganda Exp A and B while controlling for stimulation, phenotype, sex, and age. Counts were normalized for RNA composition using the trimmed mean of M-values normalization method and filtered to protein coding genes with at least 5% of libraries containing at least 1 count per million (CPM) in Uganda or 0.5 CPM in South Africa. Finally, counts were converted to log2 CPM using voom (10) and duplicates were removed, retaining libraries with the most sequences.

*Differential gene expression analysis*

We previously analyzed transcriptomes from unstimulated monocytes from South Africa and a from portion of unstimulated monocytes from Uganda (‘Exp A’, Figure 1) (5). To instead identify genes with expression patterns that distinguished RSTR and LTBI phenotypes according to the monocyte response to Mtb infection, in this study we selected an expression model that incorporated an interaction term in addition to the main effects: Expression ~ phenotype + stimulation + phenotype:stimulation + covariates with patient and genetic kinship (when available) included as random effects using R packages coxme (11) or lme4 (12) when kinship data were not available. Differentially expressed genes (DEGs) in the interaction term (FDR <0.2) were then assessed using pairwise contrasts of the four phenotype:stimulation groups (e.g. contrasting phenotypes for each stimulation condition or contrasting stimulations for each phenotype) corrected for the same covariates and random effects as the interaction model.

For Uganda, covariates included age, sex, and sequencing batch, and pairwise genetic kinship was used as a random effect. Genotypes were determined using the Illumina MEGA^EX^ array or Infinium OmniExpress BeadChip. In PLINK2 (13), SNPs present in both arrays were filtered by Hardy-Weinberg Equilibrium (P < 1E-6), minor allele frequency (MAF > 0.05), call rate (> 0.95), and linkage disequilibrium (LD R2 < 0.1 in 50 bp windows with a 5 bp slide). Using these 63812 filtered SNPs, pairwise kinship was calculated by the robust KING method for identity-by-descent (IBD) using SNPRelate v1.22.0 (14) followed by a genetic relationship matrix (GRM) using GENESIS v2.18.0 (15). For South Africa, ancestry strongly associated with the RSTR phenotype (Supplemental Table 2) and with occupational level where White/European subjects were significantly more likely to be employed in skilled (versus unskilled) positions including supervisory roles (Chi-squared *P* = 4.1E-06). Previous studies also suggest that Black African miners have higher cumulative exposure due to congregate living conditions at mine sites and migration between the mines and their homes where additional exposure likely occurs (16, 17). Accordingly, to avoid potential misclassification of RSTR subjects due to lower exposure we removed all White/European subjects (N = 6 RSTR) from subsequent analyses. Age was included as the only covariate since all participants were male, all sequencing was completed in a single batch, and the cohort included unrelated individuals (i.e. adjusting for kinship not applicable).

*STRING network analysis*

To understand biologic connectivity between each of the 260 Uganda DEGs, we used STRING v11 network analysis (18) and identified a large cluster of interconnected genes (77 genes) and five smaller networks of three or more genes (28 genes total). These genes were assessed for enrichment against Gene Ontology (GO) gene sets using Fisher’s exact test in topGO.(19) Significant gene sets (FDR < 0.05) were plotted in GO hierarchical networks, and those at branch ends below row 7 were used to color the STRING network. This reduced gene set redundancy while capturing the most specific GO terms associated with DEGs. The STRING network was also colored by mean log2 stimulation:phenotype fold change of each gene.

*Gene set enrichment analysis*

Gene set enrichment analysis (GSEA) was performed using the Molecular Signatures Database (MSigDB v7.2) (20) Hallmark and Gene Ontology (GO) collections. Fast gene set enrichment analysis (FGSEA) (21) was used to compare fold changes of all genes in phenotype:stimulation pairwise contrasts as in the DEG contrast model above. Leading-edge genes in significant GSEA results (FDR < 0.1) were compared between Uganda and South Africa to identify potential overlaps.

*Candidate gene association study*

Polymorphisms in each of the 260 DEGs including 5kb flanking regions (Ensembl build GRCh37) were identified among genotyped Ugandan RSTR (n=74) and LTBI (n=189) participants using the Illumina MEGA^EX^ array. For each of the identified 5248 SNPs, associations between genotype and clinical phenotype (RSTR cases and LTBI controls) were explored using an additive model accounting for family relationships via a penalized quasi-liklihood approximation to the generalized linear mixed model (GENESIS (15)) that was adjusted for age, sex and the first two principal components. *P* values were adjusted for multiple comparisons using the Benjamini-Hochberg false discovery rate (FDR) method.

*Data access*

Deidentified individual participant and transcriptome data collected as part of this study is available but requires approval by Data Access Committees (DACs) relevant to each study site as required by each institutional review board. To request approval, please contact Sudha Iyenhgar ([ski@case.edu](mailto:ski@case.edu)) for Uganda data and Salome Charalambous ([scharalambous@auruminstitute.org](mailto:scharalambous@auruminstitute.org)) for South Africa data. Upon approval, a letter of collaboration from the authors will be provided to the requestor. This letter of collaboration will be required to download data from the NCBI database of Genotypes and Phenotypes (dbGaP) Data Browser (<https://www.ncbi.nlm.nih.gov/gap/>) under accession 002445.v1.p1 (for Uganda) and phs002746.v1.p1 (for South Africa). These databases can also be accessed via NCBI BioProject (<https://www.ncbi.nlm.nih.gov/bioproject/>) under accession PRJNA789928.

**References (Supplementary material)**

1. Stein CM, Zalwango S, Malone LL, Thiel B, Mupere E, Nsereko M, Okware B, Kisingo H, Lancioni CL, Bark CM, Whalen CC, Joloba ML, Boom WH, Mayanja-Kizza H. 2018. Resistance and Susceptibility to Mycobacterium tuberculosis Infection and Disease in Tuberculosis Households in Kampala, Uganda. Am J Epidemiol 187:1477-1489.

2. Stein CM, Nsereko M, Malone LL, Okware B, Kisingo H, Nalukwago S, Chervenak K, Mayanja-Kizza H, Hawn TR, Boom WH. 2019. Long-term Stability of Resistance to Latent Mycobacterium tuberculosis Infection in Highly Exposed Tuberculosis Household Contacts in Kampala, Uganda. Clin Infect Dis 68:1705-1712.

3. Ntshiqa T, Chihota V, Mansukhani R, Nhlangulela L, Velen K, Charalambous S, Maenetje P, Hawn TR, Wallis R, Grant AD, Fielding K, Churchyard G. 2021. Comparing the performance of QuantiFERON-TB Gold Plus with QuantiFERON-TB Gold in-tube among highly TB exposed gold miners in South Africa. Gates Open Research 5.

4. Chihota V, Ntshiqa T, Maenetje P, Mansukhani R, Velen K, Hawn TR, Wallis RS, Grant AD, Churchyard GJ, Fielding KL. 2022. Resistance to Mycobacterium tuberculosis infection among highly TB exposed South African gold miners. PLoS One In Press.

5. Simmons JD, Van PT, Stein CM, Chihota V, Ntshiqa T, Maenetje P, Peterson GJ, Reynolds A, Benchek P, Velen K, Fielding KL, Grant AD, Graustein AD, Nguyen FK, Seshadri C, Gottardo R, Mayanja-Kizza H, Wallis RS, Churchyard G, Boom WH, Hawn TR. 2021. Monocyte metabolic transcriptional programs associate with resistance to tuberculin skin test/interferon-gamma release assay conversion. J Clin Invest 131.

6. Dobin A, Davis CA, Schlesinger F, Drenkow J, Zaleski C, Jha S, Batut P, Chaisson M, Gingeras TR. 2013. STAR: ultrafast universal RNA-seq aligner. Bioinformatics 29:15-21.

7. R_Core_Team. 2020. R: A language and environment for statistical computing. , v4.0.2. R Foundation for Statistical Computing, Vienna, Austria. <https://www.R-project.org/>.

8. Wickham H, Averick M, Bryan J, Chang W, McGowan LDA, François R, Grolemund G, Hayes A, Henry L, Hester J, Kuhn M, Pedersen TL, Miller E, Bache SM, Müller K, Ooms J, Robinson D, Seidel DP, Spinu V, Takahashi K, Vaughan D, Wilke C, Woo K, Yutani H. 2019. Welcome to the Tidyverse. Journal of Open Source Software 4:1-6.

9. Zhang Y, Parmigiani G, Johnson WE. 2020. ComBat-seq: batch effect adjustment for RNA-seq count data. NAR Genom Bioinform 2:lqaa078.

10. Law CW, Chen Y, Shi W, Smyth GK. 2014. voom: Precision weights unlock linear model analysis tools for RNA-seq read counts. Genome Biol 15:R29.

11. Therneau TM. 2020. coxme: Mixed Effects Cox Models, v2.2-16. CRAN, <https://cran.r-project.org/package=coxme>.

12. Bates D, Mächler M, Bolker B, Walker S. 2015. Fitting Linear Mixed-Effects Models Using lme4. 2015 67:48.

13. Chang CC, Chow CC, Tellier LC, Vattikuti S, Purcell SM, Lee JJ. 2015. Second-generation PLINK: rising to the challenge of larger and richer datasets. Gigascience 4:7.

14. Zheng X, Levine D, Shen J, Gogarten SM, Laurie C, Weir BS. 2012. A high-performance computing toolset for relatedness and principal component analysis of SNP data. Bioinformatics 28:3326-8.

15. Gogarten SM, Sofer T, Chen H, Yu C, Brody JA, Thornton TA, Rice KM, Conomos MP. 2019. Genetic association testing using the GENESIS R/Bioconductor package. Bioinformatics 35:5346-5348.

16. Crush J, Williams B, Gouws E, Lurie M. 2005. Migration and HIV/AIDS in South Africa. Development Southern Africa 22:293-318.

17. Hnizdo E, Murray J. 1998. Risk of pulmonary tuberculosis relative to silicosis and exposure to silica dust in South African gold miners. Occup Environ Med 55:496-502.

18. Szklarczyk D, Gable AL, Lyon D, Junge A, Wyder S, Huerta-Cepas J, Simonovic M, Doncheva NT, Morris JH, Bork P, Jensen LJ, Mering CV. 2019. STRING v11: protein-protein association networks with increased coverage, supporting functional discovery in genome-wide experimental datasets. Nucleic Acids Res 47:D607-D613.

19. Alexa A, Rahnenfuhrer J. 2021. topGO: Enrichment Analysis for Gene Ontology. <https://bioconductor.org/packages/release/bioc/html/topGO.html>. Accessed

20. Liberzon A, Birger C, Thorvaldsdottir H, Ghandi M, Mesirov JP, Tamayo P. 2015. The Molecular Signatures Database (MSigDB) hallmark gene set collection. Cell Syst 1:417-425.

21. Korotkevich G, Sukhov V, Budin N, Shpak B, Artyomov MN, Sergushichev A. 2021. Fast gene set enrichment analysis. bioRxiv doi:10.1101/060012:060012.
